# Transferrin receptor targeting by de novo sheet extension

**DOI:** 10.1073/pnas.2021569118

**Published:** 2021-04-20

**Authors:** Danny D. Sahtoe, Adrian Coscia, Nur Mustafaoglu, Lauren M. Miller, Daniel Olal, Ivan Vulovic, Ta-Yi Yu, Inna Goreshnik, Yu-Ru Lin, Lars Clark, Florian Busch, Lance Stewart, Vicki H. Wysocki, Donald E. Ingber, Jonathan Abraham, David Baker

**Affiliations:** ^a^Department of Biochemistry, University of Washington, Seattle, WA 98195;; ^b^Institute for Protein Design, University of Washington, Seattle, WA 98195;; ^c^HHMI, University of Washington, Seattle, WA 98195;; ^d^Department of Microbiology, Blavatnik Institute, Harvard Medical School, Boston, MA 02115;; ^e^Wyss Institute for Biologically Inspired Engineering at Harvard University, Boston, MA 02115;; ^f^Department of Bioengineering, University of Washington, Seattle, WA 98195;; ^g^Department of Chemistry and Biochemistry, The Ohio State University, Columbus, OH 43210;; ^h^Resource for Native Mass Spectrometry Guided Structural Biology, The Ohio State University, Columbus, OH 43210;; ^i^Department of Surgery and Vascular Biology Program, Harvard Medical School and Boston Children’s Hospital, Boston, MA 02115;; ^j^Harvard John A. Paulson School of Engineering and Applied Sciences, Harvard University, Cambridge, MA 02539;; ^k^Division of Infectious Diseases, Department of Medicine, Brigham and Women’s Hospital, Boston, MA 02115;; ^l^Broad Institute of MIT and Harvard, Cambridge, MA 02142

**Keywords:** computational protein design, drug delivery, neurological disease, transferrin receptor, blood–brain barrier

## Abstract

The de novo design of polar protein–protein interactions is challenging because of the thermodynamic cost of stripping water away from the polar groups. Here, we describe a general approach for designing proteins which complement exposed polar backbone groups at the edge of beta sheets with geometrically matched beta strands. We used this approach to computationally design small proteins that bind to an exposed beta sheet on the human transferrin receptor (hTfR), which shuttles interacting proteins across the blood–brain barrier (BBB), opening up avenues for drug delivery into the brain. We describe a design which binds hTfR with a 20 nM *K*_d_, is hyperstable, and crosses an in vitro microfluidic organ-on-a-chip model of the human BBB. Our design approach provides a general strategy for creating binders to protein targets with exposed surface beta edge strands.

## Significance

The de novo design of proteins that bind natural target proteins is useful for a variety of biomedical and biotechnological applications. We describe a design strategy to target proteins containing an exposed beta edge strand. We use the approach to design binders to the human transferrin receptor which shuttles back and forth across the blood–brain barrier. Such binders could be useful for the delivery of therapeutics into the brain.

While most protein–protein interfaces are composed primarily of sidechain–sidechain interactions, backbone hydrogen bonding can also play a role. For example, beta sheet hydrogen bonds across protein–protein interfaces are present in complexes of PDZ domains with their peptide targets, SUMO–SIM interactions, and Serpin-protease complexes among others (1, 2); in each case, the result is an extended beta sheet that spans both partners. Such backbone interactions can contribute to interaction specificity even though backbone hydrogen bonding groups are present on all residues: Formation of hydrogen bonds with the correct geometry requires precise alignment of often twisted or curved beta strands—the structure of the interacting edge strand in a binder must be matched to the structure of the edge strand it interacts with in the target. Pathological processes such as the formation of amyloid fibrils also involve beta sheet extension (3), and inhibitors that hydrogen bond to the beta sheet have been developed to block such extension (4, 5). Design approaches have been used to create homodimeric structures with extended beta sheets (6, 7) that rely on symmetrical/self-docking of scaffolds to form homodimers. But to date, methods for designing heterodimeric complexes through beta sheet extension in which one component (the target protein) is fixed have not been described, even though targets with an exposed edge strand constitute a substantial class of therapeutically interesting molecules (1, 2).

A challenge in designing binding proteins is how to form an extensive binding interface while avoiding the energetically unfavorable burial of nonhydrogen-bonded polar groups on the target. Indeed, previous de novo binder design efforts have focused primarily on sidechain–sidechain interactions with hydrophobic patches on target protein surfaces with few polar groups (8, 9). With this approach, polar regions of a target protein surface are difficult to design binders against as it is very challenging to make sidechain-mediated hydrogen bonds to all the exposed polar groups simultaneously; in particular, the many exposed C=O and N–H groups at the edges of beta sheets are difficult to fully engage with sidechain hydrogen bonds. We reasoned that designed binding proteins with edge beta strands complementary in shape to an exposed beta strand in the target protein could overcome this challenge, as the multiple strand–strand hydrogen bonds could compensate for the loss of interactions with water.

## Results

**Fig. 1.**
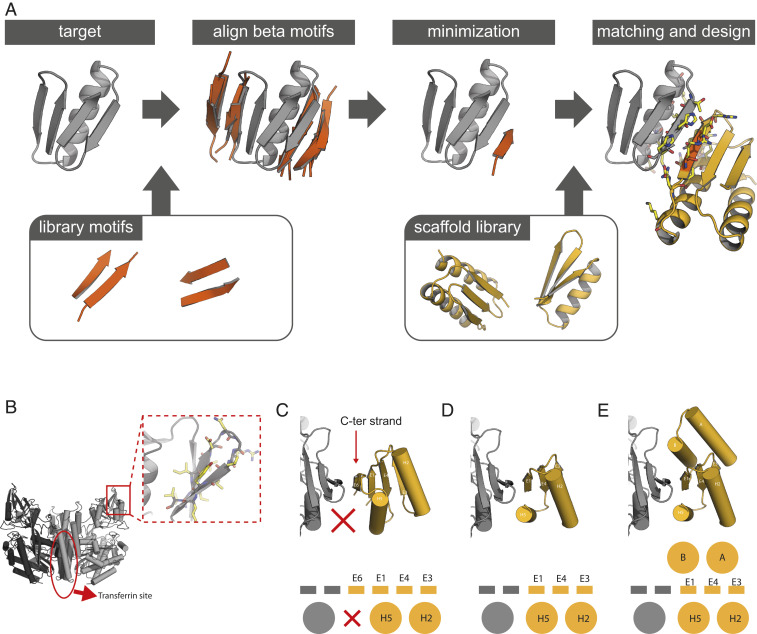
Design of edge strand–mediated complexes. (*A*) Design pipeline. After detection of exposed edge strands, a library of two-stranded beta sheet motifs was used to generate a docked strand: one of the two strands in each motif are aligned to the edge strands such that the second strand in the motif forms nonclashing beta sheet hydrogen bonds with the target that was subsequently minimized to optimize the backbone hydrogen bonds. Scaffolds from an in silico library are then superimposed or grafted onto this strand and scaffold residues are optimized to make favorable interactions with the target through the interface strand and flanking helices. (*B*) The homodimeric human transferrin receptor ectodomain [PDB: 3kas (32)] contains an exposed edge strand in the apical domain (red box). (*C*) Full-length designed ferredoxins can be docked to hTfR via strand E6, but there is little packing between helix H5 and hTfR (red X). (*D*) Strategy 1: Removal of strand E6 and instead docking via strand E1 allows for better packing interactions between helix H5 and hTfR. (*E*) Strategy 2: Expansion of the truncated scaffolds with additional helices A and B allows for an even larger burial of surface area in the interface than in strategy 1.

We developed a computational design approach for designing binding proteins with beta sheets geometrically poised to pair with exposed beta strands in target proteins of interest. We first align short two-stranded beta sheet motifs to the target protein edge strands and then use gradient-based minimization of the backbone coordinates to optimize the hydrogen bonding interactions across the interface with the target (Fig. 1*A*). These optimized beta strands are then grafted (9) onto small de novo designed protein scaffolds with geometrically matching beta sheets, yielding a docked protein–protein complex. After filtering docks based on hydrogen bond geometry and buried surface area across the interface, Rosetta flexible backbone combinatorial sequence design is used to design additional specific sidechain–sidechain interactions across the interface and to stabilize the designed scaffold.

We sought to use our protocol to design a human transferrin receptor (hTfR) binding protein. hTfR transports transferrin (the major carrier of iron in the body) across the blood–brain barrier (BBB) via receptor mediated transcytosis, and this process has been exploited to deliver therapeutic payloads into the brain parenchyma that would otherwise be blocked by the BBB (10). For example, antibodies and nanoparticles linked to larger complicated molecules such as transferrin or anti-TfR antibodies have been shown to cross the BBB into the brain parenchyma in a hTfR dependent manner (11–13). Thus, hTfR is an attractive target candidate for the development of BBB traversing vehicles (10).

We targeted the region surrounding an edge strand located in the hTfR apical domain (Fig. 1*B*). This domain is distant from the transferrin binding site (which is important to avoid competitive binding) and is exposed and therefore suitable for beta sheet extension. We first experimented with de novo–designed ferredoxins as a base scaffold for the grafting step of the protocol after strand docking, as these scaffolds contain beta sheets into which the docked strand can be grafted and helices for additional contacts to hTfR. We found that while such scaffolds could make good edge strand interactions after the grafting/matching step, the number of additional contacts between the ferredoxin helices and hTfR were limited (Fig. 1*C*). To increase the buried surface area in the interface, we followed two strategies. In strategy 1, we reasoned that truncating the ferredoxin C-terminal strand would shift the helix closer to hTfR, allowing for more extensive contacts to the target (Fig. 1*D*). In strategy 2, using RosettaRemodel (14, 15), we expanded the truncated scaffold by adding a poly valine helix at the N terminus to form a second interface with the target. Thousands of backbones were generated, in some of which the secondary interface helix was stabilized with another buttressing helix (Fig. 1*E* and *SI Appendix*, Fig. S1*A*).

**Fig. 2.**
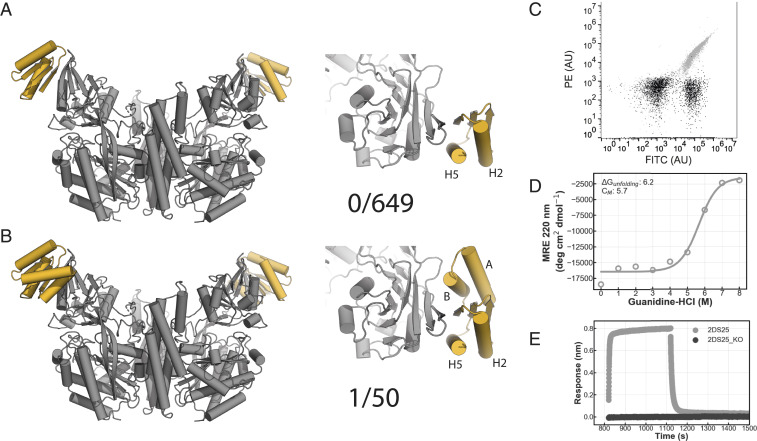
Design of a hTfR binding protein. (*A*) Model of first generation TfR binders (gray: TfR ectodomain, yellow: binder). (*B*) Model of the second generation TfR binders (gray: TfR ectodomain, yellow: binder). (*C*) 2DS25 (design: gray, negative control: black) binds to hTfR ectodomain in flow cytometry. A total of 100,000 cells were measured. (*D*) CD chemical denaturation experiment of 2DS25. (*E*) Single concentration biolayer interferometry assay (gray: 2DS25, black: 2DS25_KO [W81A/Q85A]).

A library of 649 selected designs were ordered for strategy 1 on an oligoarray, and 50 synthetic genes were ordered of the larger strategy 2 designs and tested for binding using yeast surface display (Fig. 2 *A* and *B*). Of the 649 designs from strategy 1, none bound to hTfR, despite having high in silico folding propensity and high interface shape complementarity (*SI Appendix*, Figs. S1*B* and S2). However, for strategy 2, one design (designated 2DS25) clearly bound fluorescently labeled hTfR (Fig. 2 *B* and *C*). The flaw in the strategy 1 designs was likely the still low interface buried surface area, despite truncation of the C-terminal strand of the ferredoxin scaffold. The interface buried surface area ranged from 144 to 1,395 Å^2^ but averaged only 842 Å^2^, less than that typically observed in natural complexes (16), whereas the strategy 2 designs had greater buried surface area with the target while retaining good shape complementarity (*SI Appendix*, Fig. S1*B*). In the 2DS25 design model, two helices on either side of the central beta sheet extension make contacts with TfR across the interface (*SI Appendix*, Fig. S3*A*). Binding on the yeast surface was specific as 2DS25 did not bind to the edge strand containing proteins CTLA4 and IL17 nor to polyspecificity reagents developed previously for the identification of nonspecific antibodies (*SI Appendix*, Fig. S3*B*) (17).

We next expressed and purified 2DS25 from *Escherichia coli* using immobilized metal affinity chromatography (IMAC). The protein eluted as a monodisperse peak from size-exclusion chromatography (SEC) and was confirmed to be a monomer in solution by native mass spectrometry (*SI Appendix*, Fig. S3 *C* and *D*). Circular dichroism (CD) spectroscopy showed that 2DS25 is highly stable: The melting temperature is above 95 °C, and the guanidine–HCl concentration required for 50% denaturation was 5.7 M (Fig. 2*D* and *SI Appendix*, Fig. S3 *E* and *F*). Purified 2DS25 bound the hTfR ectodomain in biolayer interferometry experiments (Fig. 2*E*). Mutation of key residues in the designed binding site abolished binding, suggesting that complex formation is through the designed interface (Fig. 2*E*). In a yeast surface competition experiment, 2DS25 competed for binding with Machupo virus (MACV) GP1, a viral glycoprotein which binds the TfR apical domain, suggesting 2DS25 binds the targeted area on hTfR (*SI Appendix*, Fig. S3*G*).

To probe the sequence determinants of folding and binding, and to facilitate determination of the structure of the 2DS25–hTfR complex, we created a site saturation mutagenesis (SSM) library in which each position on 2DS25 was substituted with all other 20 amino acids one at a time and screened for hTfR binding using fluorescence-activated cell sorting (*SI Appendix*, Fig. S4). Deep sequencing revealed that the designed core residues of 2DS25 were conserved, suggesting 2DS25 folds as designed. The key interface residues were also conserved, while affinity-increasing substitutions were identified around the interface. Combination of these enriched substitutions yielded higher affinity variants (see *Materials and Methods*).

**Fig. 3.**
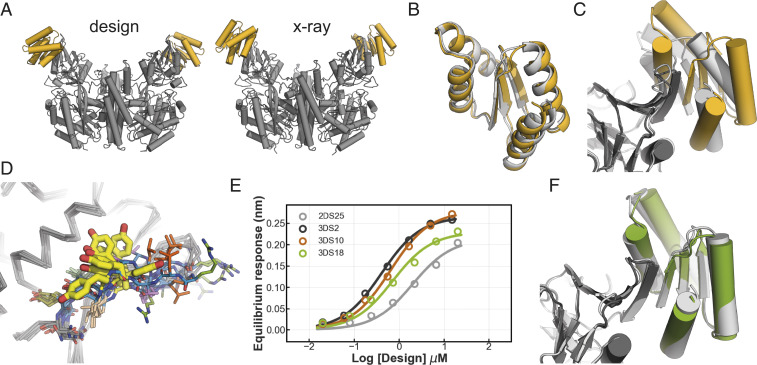
Structural analyses of 2DS25 complexes. (*A*) Overview of the designed model and crystal structure (gray: hTfR, yellow: 2DS25). (*B*) Superposition 2DS25.5 (yellow) and the designed model (light gray). (*C*) Superposition of the 2DS25.5-hTfR crystal structure (dark gray and yellow) and designed model (light gray) at the interface. (*D*) Overlay of hTfR ectodomain structures in the PDB (1cx8, 1de4, 1suv, 3kas, 6d04, 3s9l, 6h5i, 6wrv, and 6wrv). Edge strand backbones are colored in blue. Tyr211 is shown in thick yellow sticks. (*E*) Equilibrium binding curves 2DS25 and the designs 3DS2, 3DS10, and 3DS18. (*F*) Overlay of the crystal structure 3DS18 in complex with hTfR (green and dark gray) and the designed model (light gray).

The crystal structures of two increased affinity variants (2DS25.1 and 2DS25.5, *SI Appendix*, Table S2) in complex with hTfR were determined to be resolutions of 3.1 and 2.8 Å, respectively. The structures are virtually identical with an rmsd of 0.26 Å (*SI Appendix*, Fig. S5). In both cases, the design binds the Apical domain of hTfR using beta sheet extension and overall closely resembles the computational design model (Fig. 3*A* and *SI Appendix*, Table S1). The structure of 2DS25 superimposes closely on the computational model with an rmsd of 1.2 Å (Fig. 3*B*); this is notable as the fold is more complex than that of previous computationally designed binders. Closer inspection of the interface shows that the actual beta sheet register in the crystal structure is shifted compared to the computational design model (Fig. 3*C* and *SI Appendix*, Figs. S6*A* and S7*A*), likely due to differences in the structure of the TfR apical region compared to that in the structure (Protein Data Bank [PDB]: 3kas) used in the design calculations (*SI Appendix*, Fig. S6*B*). The structural differences in the receptor are at both the backbone and side chain level, in particular at Tyr211; a superposition of all hTfR apo and holo structures in the PDB shows this region is flexible and can exist in multiple conformations (Fig. 3*D*).

As the conformation of hTfR observed in our crystal structure differs from those in other structures of the receptor, we investigated what our design protocol produced when targeted against this new conformation. In this new design round, we also loosened constraints of χ_2_ torsion angles of aromatic residues normally imposed during design calculations to allow for more strained but overall favorable π–π interactions present in the crystal structure (*SI Appendix*, Figs. S7 and S8). We selected 48 designs and expressed them in *E. coli*. Of the 48 designs ordered, 24 were soluble after SEC, and 7 designs showed a binding signal in biolayer interferometry (*SI Appendix*, Fig. S9*A*), a substantial improvement in success rate compared to the previous design round. We proceeded with three designs for further biophysical characterization and found that they bound with affinities ranging from 400 to 700 nM (Fig. 3*E* and *SI Appendix*, Figs. S9*B*, S13 and Table S2). The 2.5 Å resolution crystal structure of design 3DS18 in complex with hTfR very closely matches the computational model (Fig. 3*F* and *SI Appendix*, Fig. S10 *A* and *B*). The docked complexes of 3DS18 and 2DS25 are similar on the backbone level, but apart from the side chains in the docked-strand edge strand, none of the interface residues are the same (*SI Appendix*, Fig. S10 *C* and *D*).

### 2DS25 Crosses the BBB In Vitro.

**Fig. 4.**
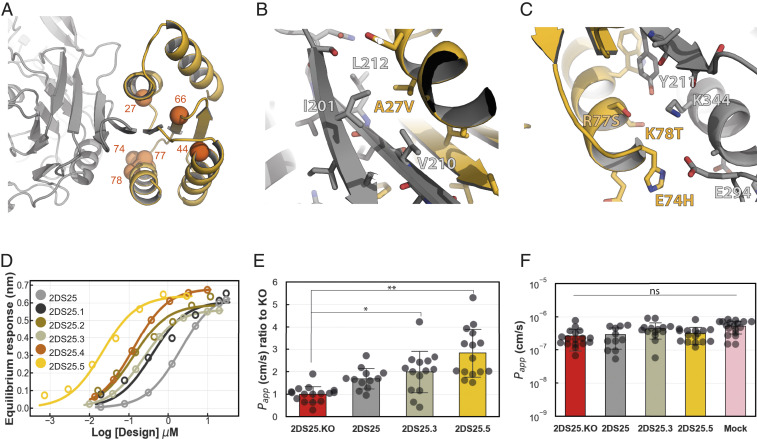
In vitro BBB traversal of 2DS25 variants. (*A*) Positions on 2DS25 that improve binding (C-alpha atoms as orange spheres). (*B*) The A27V substitution was highly enriched in site saturation mutagenesis and creates a snug packing interaction with a hydrophobic patch on hTfR. (*C*) On the opposite side of the interface, E74H, R77S, and K78T may improve electrostatic complementarity. (*D*) Biolayer interferometry equilibrium binding curves of 2DS25 and optimized variants. (*E*) Transcytosis of 2DS25 variants in the in vitro human BBB chip model. Measurement is taken after 3 h. *P value 0.033, **P < 0.0001, Dunnett’s test. (*F*) Barrier integrity of the BBB chips at 3 h. Bar plots show mean values with SD as error bars.

Binding affinity is a key factor determining transcytosis efficiency of compounds targeting hTfR. For instance, monovalent or low-affinity antibodies were found to transcytose more efficiently than higher affinity antibodies, which instead were targeted for lysosomal degradation (11, 12, 18), suggesting an optimal *K*_*D*_ exists for transcytosis. We hence took advantage of the SSM data to create a range of designs with different *K*_*D*_’s (see *Materials and Methods*). The majority of the mutants that improved binding map to the interface between hTfR and 2DS25 and likely optimize packing interactions and electrostatic contacts (Fig. 4 *A*–*C* and *SI Appendix*, Fig. S11*A*). Two mutants (A44G and I66L) that improved binding occurred in the core of 2DS25 distal to the interface; these may produce subtle conformational alterations that stabilize the interface (*SI Appendix*, Fig. S11*B*). Biolayer interferometry of five variants revealed *K*_*D*_’s ranging from 20 to 400 nM (Fig. 4*D* and *SI Appendix*, Figs. S12 and S13 and Table S2).

We explored the potential of our designs for BBB traversal using a recently developed human in vitro BBB model that was created using microfluidic organ chip technology (19). These BBB chips contain two parallel microfluidic channels separated by a porous extracellular matrix-coated membrane; the membrane is lined by human-induced pluripotent stem cell–derived brain microvascular endothelial cells (iPS-BMVECs) on one side, creating a “vascular” channel and primary human brain pericytes and astrocytes on the other side of the membrane, creating the central nervous system (CNS) channel. These chips recapitulate key features of the human BBB including a permeability barrier similar to that observed in vivo, the expression of physiologically relevant multiple drug transporters and hTfRs that can recapitulate shuttling of anti-TfR antibodies across the BBB in vitro (19) and undergo in vivo-like transcytosis of brain-seeking extracellular vesicles in vitro (20). Following injection of three Alexa488 labeled designs spanning two orders of magnitude in *K*_D_ (2DS25 2 μM, 2DS25.3 200 nM, and 2DS25.5 20 nM) into the vascular channel, we observed transport of 2DS25.3 and 2DS25.5 into the CNS channel (Fig. 4*E*). Transport across the BBB was slightly higher for 2DS25.5 (*K*_d_ 20 nM) compared to 2DS25.3 (*K*_d_ 200 nM). An interface knockout (KO) variant of 2DS25 showed lower penetration into the CNS channel than 2DS25.3 and 2DS25.5, indicating the transport was specific. Transport of the base design 2DS25 was comparable to the interface KO, suggesting that the low *K*_d_ (2 μM) hampers transport into the CNS channel. In all performed assays, barrier integrity was maintained throughout the experiment (Fig. 4*F*).

## Discussion

Our method for computationally designing small proteins that bind to exposed beta strands and neighboring regions on protein targets considerably expands the possibilities for protein inhibitor design. “One-sided” interface design in which a protein is de novo designed to bind to a fixed target protein with high specificity and affinity has been largely limited until now to targets with surface hydrophobic patches, which can be complemented by appropriately shaped hydrophobic clusters on the designed protein. Our method now makes available the much more polar and less concave regions surrounding edge beta strands and hence increases the number of proteins of interest which can be targeted.

The relative orientation of the edge strand and flanking helices on our designed scaffolds were important for success. Truncation of the base ferredoxin scaffold and addition of helices yielded scaffolds with improved binding metrics when docked. Inspection of other edge strand–containing targets suggests that the overall structural context of the target edge strand is often similar to that in hTfR, with a central edge strand flanked by helices albeit with relative orientations that are different from in hTfR. Exhaustive sampling of the positions of helices relative to the edge strand on a designed scaffold should give rise to a family of scaffolds that can engage a large number of edge strand–containing target proteins.

The advantage of computational design over antibody and other selection methods in being able to choose the region of the target being bound is clear in the hTfR case; we selected a site far away from the transferrin binding site to avoid competition. The observation of strand shifting in the target structure highlights the importance of targeting regions which are relatively immobile; in the case of hTfR, a superposition of the available structures indicates high mobility at the edge strand, which we failed to take into account in the first design rounds. Moving forward, design against multiple target crystal structures or conformations produced by molecular dynamics simulations and other methods may be useful, particularly in cases in which multiple crystal structures are not available.

Our results on the in vitro BBB traversal of 2DS25.3 and 2DS25.5 are encouraging, but it must be emphasized that for more detailed characterization of the BBB traversal properties of our designs, in vivo experiments beyond the scope of this study will be required. Assessment of the kinetics of BBB traversal both in vitro and in vivo as well as comparison against current state of the art BBB shuttling systems (21, 22) will be important to evaluate the full potential of our designs for BBB traversal. Once further characterized, our small, stable designed hTfR binder, and similar designs against other targets at the BBB may provide new possibilities for transporting therapeutics and other molecular cargo into the brain. The small size (10 kDa) and different binding mode to hTfR may offer improved access to the brain compared to antibodies and the cognate ligand transferrin (which is 76 kDa). Given the high stability and modularity, and likely robustness to genetic fusion and chemical coupling, our designs could have a distinct advantage over larger more complicated molecules for fusion/coupling to therapeutic cargoes.

## Materials and Methods

### Protein Design.

#### Identification of target edge strands.

The transferrin receptor target protein (PDB: 3kas) was relaxed in the Rosetta energy function using coordinate constraints after removing hetero atom records. All target protein edge strands were identified visually by inspection in a molecular graphics viewer or programmatically by calculating the atomic solvent accessible surface area (aSASA) of all backbone H and O atoms present in residues that were in beta conformation. Strands with a length of at least three residues and an average aSASA value above 2 were considered solvent exposed and hence edge strands suitable for strand docking.

#### Geometric matching beta motifs to edge strands.

The C-alpha atoms of short parallel and antiparallel two-stranded beta sheets derived from the PDB were aligned onto the target edge strand. The aligned segments of the motifs were next deleted. The docked strands were then either trimmed down further or extended at either the N or C terminus, creating a range of strands with different lengths. These docks were relaxed using gradient descent–based minimization in the presence of the target using Rosetta FastRelax to optimize backbone hydrogen bond interactions with the target edge strand. Docks failing a specified threshold value (typically −4) for the backbone hydrogen bond score term in Rosetta (hbond_lr_bb) were discarded.

#### Matching docked and minimized strands into scaffolds.

Strands were geometrically matched using the MotifGraftMover (9) in Rosetta (see *SI Appendix* for xml with all parameters) to a scaffold library consisting of ferredoxin-like scaffolds and modified ferredoxin-like scaffolds expanded with additional helices. Following matching, the resulting protein–protein complexes were repacked at the interface using the PackRotamersMover followed by cartesian and kinematic (FastRelax) minimization to regularize the potentially broken bonds at the junctions of the docked strand and the scaffold.

#### Interface design and filtering.

The interface side chains of the complexes were designed using Rosetta combinatorial sequence optimization with as score function “ref2015,” “beta_nov16,” or “beta_genpot” to minimize the sidechain–sidechain interaction energy and maximize the stability of the designed scaffolds (see *SI Appendix* for example design RosettaScripts). During sequence optimization, the backbones of the designed scaffolds were allowed to move, enabling finer sampling of the possible side chains. In addition, rigid body minimization was allowed during the design protocol.

In general, the best designs in terms of interface energy per buried surface area (≤−25 Rosetta energy units [REU]), interface shape complementarity (≥0.6), interface buried surface area (≥1,200 Å^2^), average per residue energy (≤−2 REU), and the number of buried unsatisfied polar in atoms in the interface (≤3) were inspected visually before selecting designs for ordering as synthetic genes. As an additional filtering step, multiple independent Rosetta folding simulations were performed to assess whether our designed sequences would fold into the lowest energy structures without deep off-target minima.

#### Backbone generation and scaffold design.

De novo designed ferredoxin-like scaffolds that served as the basis for the first hTfR binders, were modified, and expanded using blueprint-based backbone generation (14, 23). Backbone generation was biased to only include idealized canonical loops to connect secondary structure elements (23). Rosetta combinatorial sequence optimization was used to design the sequence of the new backbones (15, 24–28). Low-energy designs that folded into the designed structure in Rosetta folding simulations were selected and used as scaffolds for hTfR binders.

### Protein Purification and Expression.

Synthetic genes encoding designed proteins and their variants were purchased from Integrated DNA Technologies or Genscript (see the spreadsheet in *SI Appendix* for detailed construct information). Sequences included N-terminal histidine tags followed by a tobacco etch virus (TEV) cleavage site. All genes were expressed by autoinduction in thyrotropin binding inhibiting immunoglobulins media (MP Biomedicals) supplemented with 50 × 5,052, 20 mM MgSO_4_, and trace metal mix. Expression was allowed under antibiotics selection at 37 C° overnight or at 18 to 25 C° overnight after initial growth for 6 to 8 h at 37 C°.

Next, cells were harvested by centrifugation and lysed by sonication after resuspension of the cells in lysis buffer (100 mM Tris pH 8.0, 200 mM NaCl, 50 mM Imidazole pH 8.0) containing protease inhibitors (Thermo Scientific) and bovine pancreas DNaseI (Sigma-Aldrich). Proteins were subsequently purified by IMAC. Cleared lysates were applied to 2 to 4 mL nickel NTA beads (Qiagen) and incubated in batch for 20 min before washing beads with 10 to 20 column volumes of lysis buffer. Designs were eluted in elution buffer (20 mM Tris pH 8.0, 100 mM NaCl, 500 mM Imidazole pH 8.0) after which the histidine tags were cleaved using histidine-tagged TEV protease, while dialyzing against dialysis buffer overnight (20 mM Tris pH8.0, 100 mM NaCl). A second IMAC purification was performed the next day for TEV-cleaved samples to capture uncleaved protein and TEV protease. Designs were finally polished using SEC on either Superdex 200 Increase 10/300GL or Superdex 75 Increase 10/300GL columns (GE Healthcare) using SEC buffer (10 mM Hepes pH 7.5, 100 mM NaCl). Peak fractions were verified by SDS-polyacrylamide gel electrophoresis and liquid chromatography with mass spectrometry and stored at concentrations between 1 to 10 mg/mL at 4 C° or flash frozen in liquid nitrogen for storage at −80. MACV GP1 (Carvallo strain, NC_005078, residues 87 to 240) containing an N-terminal histidine tag, a TEV protease site, and a short linker (amino acids SGSG) was produced and purified as previously described (29).

The hTfR 1 ectodomain (uniprot P02786-1) was expressed as a fusion protein (IgK-sFLAG-His-Scn-TEV-TfR1-his-Avi, see the spreadsheet in *SI Appendix* for detailed construct information) using the Daedalus expression system (30). After cleaving the N-terminal expression tag with TEV, the protein was further purified by SEC. Peak fractions were biotinylated using an in vitro biotinylation kit (Avidity). Biotinylated TfR was further purified by Superdex 200 Increase 10/300GL in SEC buffer. Peak fractions were concentrated to ∼1.5 mg/mL, flash-frozen, and stored at −80 C°.

For structural studies, a soluble fragment of the hTfR 1 ectodomain (residues 121 to 760) was cloned into the pHLsec expression vector (31). We produced TfR1 in human embryonic kidney 293S GnTI^−/−^ cells (ATCC CRL-3022) grown in suspension culture and maintained in serum free medium (Freestyle 293 Expression Medium, Life Technologies) supplemented with 2% (volume/volume) Ultralow IgG fetal bovine serum (Thermo Fisher) and penicillin/streptomycin. Cells were transfected using polyethylenimine, and supernatant was harvested 72 h posttransfection. The hTfR was purified using human transferrin affinity chromatography as previously described (32) and further purified by SEC using Superdex 200 Increase 10/300GL column (GE Healthcare) in buffer containing 25 mM Tris⋅HCl pH 7.5, 150 mM NaCl.

### CD.

CD spectra were recorded on a J-1500 instrument (Jasco) in a 1 mm path length cuvette at a protein concentration of 0.32 mg/mL (chemical melts) or 0.4 mg/mL (temperature melts). For temperature melts, data were recorded at 220 nm between 25 and 95 °C every 2 C°, and wavelength scans (190 to 260 nm) were recorded every 10 °C in Dulbecco’s phosphate-buffered saline buffer (Gibco). Chemical denaturation wavelength scans were recorded between 190 to 260 nm in the presence of guanidine–HCl buffer at 25 °C. Data recorded at 220 nm during the chemical denaturation melts were fitted to the following model (33) using custom python scripts to obtain the m-value, Δ*G*_*0*_, *S*_*N*_, *S*_*D*_, and midpoint of denaturation value (*C*_*M*_).

$$fD=1/1+e^{\frac{m\left[denaturant\right]-\Delta G_{0}}{RT}},$$

$$S=S_{N}+\left(S_{D}-S_{N}\right)fD,$$

where *S* is the observed signal, *S*_*N*_ the signal of the folded baseline, and *S*_*D*_ the signal of the denatured baseline. *C*_*M*_ was obtained by

$$C_{M}=\frac{\Delta G_{0}}{m}.$$

### Library Generation.

The gene library for the first generation hTfR binders was ordered from Agilent Technologies with flanking adaptor sequences to allow amplification of the genes. qPCR using Kapa HiFi Hotstart Ready Mix (Kapa Biosystems) was performed to amplify the library in order to prevent overamplification that would reduce transformation efficiency. After amplification and DNA gel electrophoresis, DNA was purified using a gel extraction kit (Qiagen) and subjected to a second qPCR amplification round to add pETCON adaptors to both DNA ends to facilitate cloning into the yeast surface display vector pETCON. This gene pool was again purified by gel extraction.

The 2DS25 SSM library was generated by overlap extension PCR at each codon of the 2DS25 gene. Randomized primers were purchased from Integrated DNA Technologies. After verification of the desired inserted size by DNA gel electrophoresis, a second PCR was performed to add pETCON adaptors to both DNA ends to facilitate cloning. For both libraries, EBY100 electrocompetent yeast cells were transformed by electroporation with the linear library DNA together with the linearized (NdeI/XhoI) pETCON yeast surface display vector as described earlier (34).

### Yeast Surface Display and Deep Sequencing.

Myc-tagged designs were displayed on the yeast surface as Aga2p fusion proteins. The diversity of the libraries was below 10^6^ in all cases. Yeast cells were grown at 30 °C in C-trp-ura+2% glucose media for 16 to 24 h before expression was induced by transferring cells to synthetic galactose medium supplemented with casamino acids media (SGCAA) for 16 to 24 h at 30 °C. Cells were harvested by centrifugation and washed twice with PBSF (PBS supplemented with 1% bovine serum albumin). Cells were subsequently incubated with biotinylated hTfR for 0.5 to 2 h at room temperature before being washed twice with PBSF. These cells were next labeled with streptavidin–phycoerythrin (PE) and an FITC conjugated anti-Myc antibody (Immunology Consultants Laboratory) for 20 min before being washed again. For initial screening for binding signals, biotinylated hTfR was preincubated with streptavidin–PE (Invitrogen) for 10 min before the complex was added to cells enabling the identification of weak binders by using avid binding conditions. Samples were sorted or measured in a Sony SH800 cell sorter or Accuri flow cytometer (BD Biosciences) using the FITC and PE signals. Sorted cells were collected and grown in C-trp-ura+2% glucose media for 24 to 48 h before being frozen at −80 °C for later analyses. SSM libraries were selected against 100 nM, 20 nM, and 7 nM hTfR, whereas the combination libraries were selected against 250 nM, 10 nM, 1 nM, 0.5 nM, 0.250 nM, and 0.125 nM hTfR.

DNA preparation for deep sequencing was performed as described before (35). DNA was sequenced using MiSeq sequencer with a 600-cycle reagent kit (Illumina). Reads were aligned with paired-end read merger software (36). Sequences were finally analyzed using custom scripts based on the Enrich software (37).

### Combination Variants Generation.

After deep sequencing analyses of the SSM library, we identified 13 positions in which individual mutations improved binding. Two approaches were followed to further optimize the binding affinity. First, a subset of selected mutants was manually combined and ordered as synthetic genes for testing in binding assays. This approach yielded 2DS25.3.

In the second approach we generated a combination library. We ordered two overlapping Ultramer oligonucleotides (Integrated DNA Technologies) containing degenerate codons for the 13 specified positions. Ultramer fragments were assembled and PCR amplified before being electroporated as described above. After selecting the best binders in yeast surface display by sanger sequencing, designs were ordered as synthetic genes and purified for testing in biolayer interferometry binding assays.

Surprisingly, high-affinity variants on the yeast surface only bound with moderate affinity in the biolayer interferometry assays. Even though the off-rate decreased in these variants, this decrease was generally accompanied by a compensatory decrease in on-rate. In order to create high affinity variants with fast on-rates and slower off-rates, we manually combined positions of the SSM, 2DS25.3, and combination library mutants yielding 2DS25.5.

### Biolayer Interferometry.

Binding assays were performed on an OctetRED96 BLI system (ForteBio) using streptavidin-coated biosensors. Biosensors were equilibrated for at least 10 min in Octet buffer (10 mM Hepes pH 7.4, 150 mM NaCl, 3 mM EDTA, 0.05% Surfactant P20) supplemented with 1 mg/mL bovine serum albumin (SigmaAldrich). For each experiment, the biotinylated hTfR ectodomain was immobilized onto the biosensors by dipping the biosensors into a solution with 10 to 50 nM hTfR for 200 to 500 s, followed by dipping in fresh octet buffer to establish a baseline for 200 s. Titrations were executed at 25 °C while rotating at 1,000 rpm. Association of designs to TfR on the biosensor was allowed by dipping biosensors in solutions containing designed proteins diluted in octet buffer for 900 s. After reaching equilibrium, the biosensors were dipped into fresh buffer solution in order to monitor the dissociation kinetics for 900 to 1,500 s. In single concentration assays, 1 μM design was used diluted in octet buffer. For equilibrium binding titrations, kinetic data were collected and processed using a 1:1 binding model to obtain the equilibrium binding response Req using the data analysis software 9.1 of the manufacturer. Multiple binding experiments with different protein preparations under different hTfR immobilization densities were performed to ensure reproducibility. Representative binding curves are presented in the main text. Both global kinetic fitting using lower concentration data and steady-state saturation fits using data from all concentrations were performed for *K*_*D*_ calculations. For steady-state fits, in each design, seven Req values were fitted with a custom Python script to a saturation binding curve to obtain *B*_*max*_ and the equilibrium dissociation constant *K*_*D*_.

$$Y=\frac{B_{max}\cdot X}{K_{D}+X}.$$

### In Vitro Human BBB Chip Traversal Assays.

Single cysteine variants (E3C) of 2DS25, 2DS25_KO, 2DS25.3, and 2DS25.5 used in the human BBB chip studies were expressed and purified as described above in the presence of reducing agent TCEP. Proteins were labeled with Alexa Fluor 488 C5 maleimide (Thermo Fisher) and purified according to the manufacturer’s protocol. In vitro human BBB chips were generated as previously described (19). The human-induced pluripotent stem (iPS) cell line IMR90-C4 (WiCell Research Institute) was differentiated into human iPS-BMVECs utilizing a hypoxia-induced differentiation protocol for mimicking the embryological developmental conditions for obtaining high expression of functional tight and adherens junctions, as well as efflux proteins and hTfR.

Two-channel microfluidic devices (obtained from Emulate Inc.) were activated with Sulfo-SANPAH (Thermo Fisher) treatment prior to coating the channels with collagen IV (400 μg/mL; Sigma Aldrich) and fibronectin (100 μg/mL; Sigma Aldrich) overnight. Both channels of the chip were rinsed with PBS and then with astrocyte medium before seeding cells. Primary human astrocytes (ScienCell) and pericytes (ScienCell) were cocultured in the brain channel of the BBB chip by mixing together 0.7 × 10^6^ cells/mL astrocytes and 0.3 × 10^6^ cells/mL pericytes in the astrocyte medium, seeding them in the apical channel of the chip, and then incubating them under static conditions at 37 °C. After 1 h, unattached astrocyte and pericytes were removed by washing both channels of the chip with endothelial cell (EC) medium containing fibroblast growth factor (20 ng/mL; R&D Systems) and retinoic acid (RA) (10 μM; Sigma Aldrich) (EC+RA), and then 20 μL iPS-BMVECs (2.3 × 10^7^ cells/mL) were seeded in the basal channel, and the device was immediately placed upside down to allow the BMVECs to adhere to the matrix-coated porous membrane. After overnight incubation under static conditions at 37 °C, the chip was placed right side up and both channels of the BBB chip replaced with EC medium without growth factors were cultured under static conditions for one additional day. The next day (2 d after seeding) the BBB chips were attached to specialized chip holders with medium reservoirs (Pods; Emulate Inc.) to provide a source of EC medium to flow through both channels (60 μL/h) using the automated control features of the Zoë Culture Instrument (Emulate Inc.).

On the third day after seeding, Alexa Fluor 488 labeled 2DS25 variants at 400 nM in EC medium were flowed through the vascular channel of the BBB chip at 60 μL/h. All samples included 50 μg/mL cascade blue molecule (Thermo Fisher) to simultaneously monitor the barrier integrity of the chips during the experiments. Effluent samples were collected from both vascular and CNS channels at 3 h. Fluorescent intensity of the samples were measured, and their concentrations were calculated based on the standard curve for each compound. Apparent permeability (P_app_) values of the Alexa Fluor 488 labeled 2DS25 variants were calculated using the concentration of the protein that penetrated into the brain channel.

### Native Mass Spectrometry.

Sample purity, integrity, and oligomeric state was analyzed by on-line buffer exchange MS using a Vanish ultra-performance liquid chromatography coupled to a Q Exactive ultra-high mass range Orbitrap instrument (Thermo Fisher Scientific). A total of 5 pmol protein (0.1 μL of 50 μM protein in 10 mM Hepes pH7.5, 100 mM NaCl) were injected and on-line buffer exchanged to 200 mM ammonium acetate, pH 6.8 (AmAc) by a self-packed buffer exchange column (P6 polyacrylamide gel, BioRad) at a flow rate of 100 μL per min (38). Mass spectra were recorded for 1,000 to 8,000 *m/z* at 12,500 resolution as defined at 400 *m/z*. The injection time was set to 200 ms. Voltages applied to the transfer optics were optimized to allow ion transmission, while minimizing unintentional ion activation. Mass spectra were deconvoluted with UniDec version 4.2.0 (39).

### Structure Determination.

For all structures, starting phases were obtained by molecular replacement using Phaser (40). Diffraction images were integrated using XDS (41) and merged/scaled using Aimless (42). Structures were refined in Phenix (43) using phenix.autobuild and phenix.refine or Refmac (44). Model building was performed using COOT (45). We used SBGrid-supported applications to complete our structural studies (46).

Proteins were crystallized using the vapor diffusion method. To crystallize 2DS25.1, 2DS25.5, or 3DS18 in complex with hTfR, proteins were purified by SEC separately and mixed in a 1:1.2 molar ratio (hTfR:design) to a final concentration of 10 mg/mL. Crystals of the 2DS25.1/hTfR complex grew in 0.1 M Hepes pH 7.5 and 12% (wild type/volume) polyethylene glycol 3,350 and were flash frozen in well solution containing 20% (volume/volume) glycerol. Crystals of the 2DS25.5/hTfR complex grew in 0.1 M BICINE pH 8.5 and 8% (weight/volume) polyethylene glycol monomethyl ether 550 and were flash frozen in well solution containing 20% (weight/volume) glycerol. Crystals of the 3DS18/hTfR complex grew in 1.1 M Sodium Malonate pH 7.0, 0.1 M Hepes pH 7.0, and 0.5% Jeffamine ED-2001 pH 7.0 and were flash frozen in mother liquor. Diffraction data for the complexes were collected at a wavelength of 0.979 and temperature of 100 K on Northeastern Collaborative Access Team Advanced Photon Source (APS) beamlines 24-ID-C and 24-ID-E (APS, Argonne National Laboratory). Structures were determined by molecular replacement using PHASER with coordinates for TfR1 (PDB: 3kas) and coordinates for models of the respective designs as search models.

## Supplementary Material

Supplementary File

## Data Availability

Crystal structures have been deposited in the RCSB PDB with the accession nos. 6WRX, 6WRW, and 6WRV. Additional supporting data has been deposited in the online Zenodo repository (https://zenodo.org/record/4594115) (47). All other study data are included in the article and/or supporting information.
