# Supplementary material for: Transferrin receptor targeting by de novo sheet extension
Source: Proc Natl Acad Sci U S A. 2021 Apr 20;118(17):e2021569118. doi: 10.1073/pnas.2021569118 (PMC8092486; doi:10.1073/pnas.2021569118)
Supplement: Supplementary File [file pnas.2021569118.sapp.pdf]

Supporting Information

Transferrin receptor targeting by *de novo* sheet extension

Danny D. Sahtoe[1,2,12], Adrian Coscia[3†], Nur Mustafaoglu[4†], Lauren M. Miller[1,2, †], Daniel Olal[3], Ivan Vulovic[1,2], Ta-Yi Yu[2,5], Inna Goreshnik[1,2], Yu-Ru Lin[1,2], Lars Clark[3], Florian Busch[6,7], Lance Stewart[1,2], Vicki H. Wysocki[6,7], Donald E. Ingber[4,8,9], Jonathan Abraham[3,10,11,*] and David Baker[1,2,12,*].

[1]Department of Biochemistry, University of Washington, Seattle, WA 98195, USA.
[2]Institute for Protein Design, University of Washington, Seattle, WA 98195, USA.
[3]Department of Microbiology, Blavatnik Institute, Harvard Medical School, Boston, MA 02115, USA.
[4]Wyss Institute for Biologically Inspired Engineering at Harvard University, Boston, MA 02115, USA.
[5]Department of Bioengineering, University of Washington, Seattle, WA 98195, USA.
[6]Department of Chemistry and Biochemistry, The Ohio State University, Columbus, OH, USA.
[7]Resource for Native Mass Spectrometry Guided Structural Biology, The Ohio State University, Columbus, OH, USA.
[8]Department of Surgery and Vascular Biology Program, Harvard Medical School and Boston Children's Hospital, Boston, MA 02115, USA.
[9]Harvard John A. Paulson School of Engineering and Applied Sciences, Cambridge, MA 02539, USA.
[10]Department of Medicine, Division of Infectious Diseases, Brigham and Women's Hospital, Boston, MA 02115, USA.
[11]Broad Institute of Harvard and MIT, Cambridge, MA 02142, USA.
[12]Howard Hughes Medical Institute, University of Washington, Seattle, WA, USA
*Correspondence: David Baker dabaker@uw.edu, Jonathan Abraham jonathan_abraham@hms.harvard.edu
†Equal contribution

Supplementary Figure 1

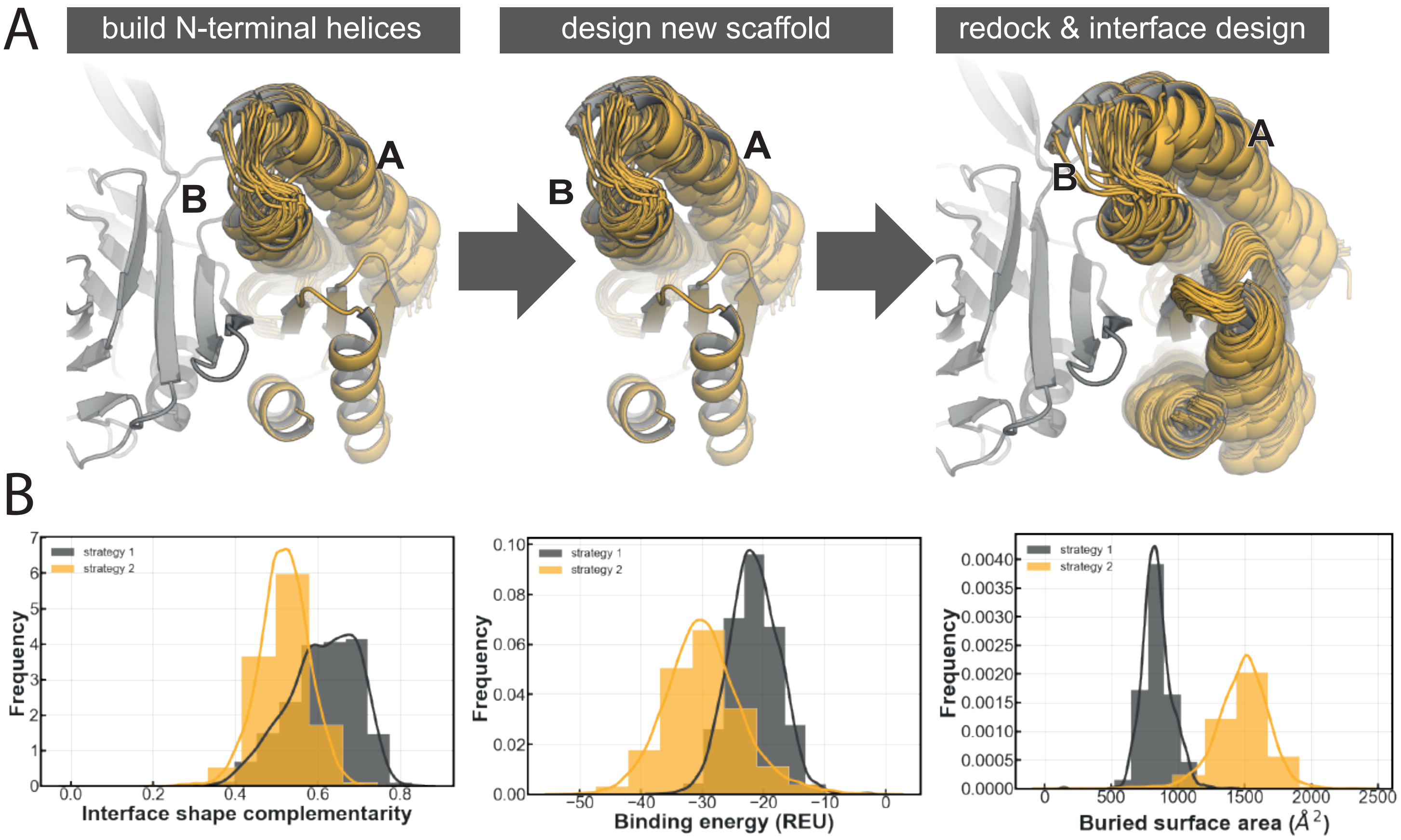

Fig S1. Design strategy2 hTfR binders. A) A second interface was build to the backside of the truncated starting scaffold to increase the interface buried surface area. Helix B was generated to make direct contacts to the target. The majority of the new designs also incorporated A as buttressing helix to stabilize helix B. The sequences of the new backbones were optimized and the best designs were redocked to the target after which the interface residues were again optimized. **B)** Histograms of computational metrics of the strategy 1 and strategy 2 designs.

Supplementary Figure 2

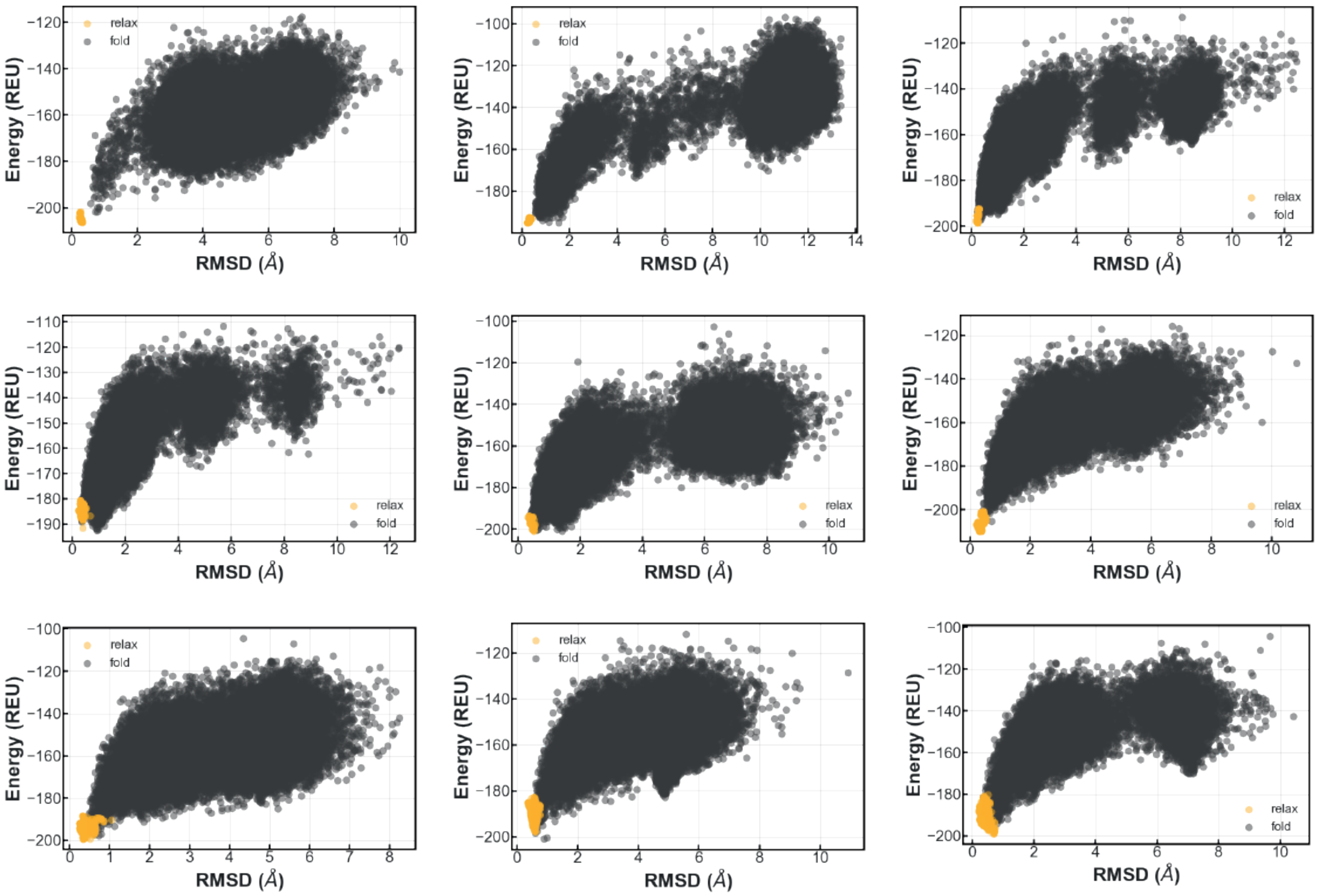

Fig S2. In silico folding of strategy 1 hTfR designs. Example energy landscapes from Rosetta folding simulations of representative designs of the strategy 1 hTfR binders. Gray dots represents lowest energy structures from independent Monte Carlo folding simulations starting from an extended chain. Yellows dots, are lowest energy structures obtained from Rosetta FastRelax simulations starting from the designed model. In general, most simulations converged onto the designed model.

Supplementary Figure 3

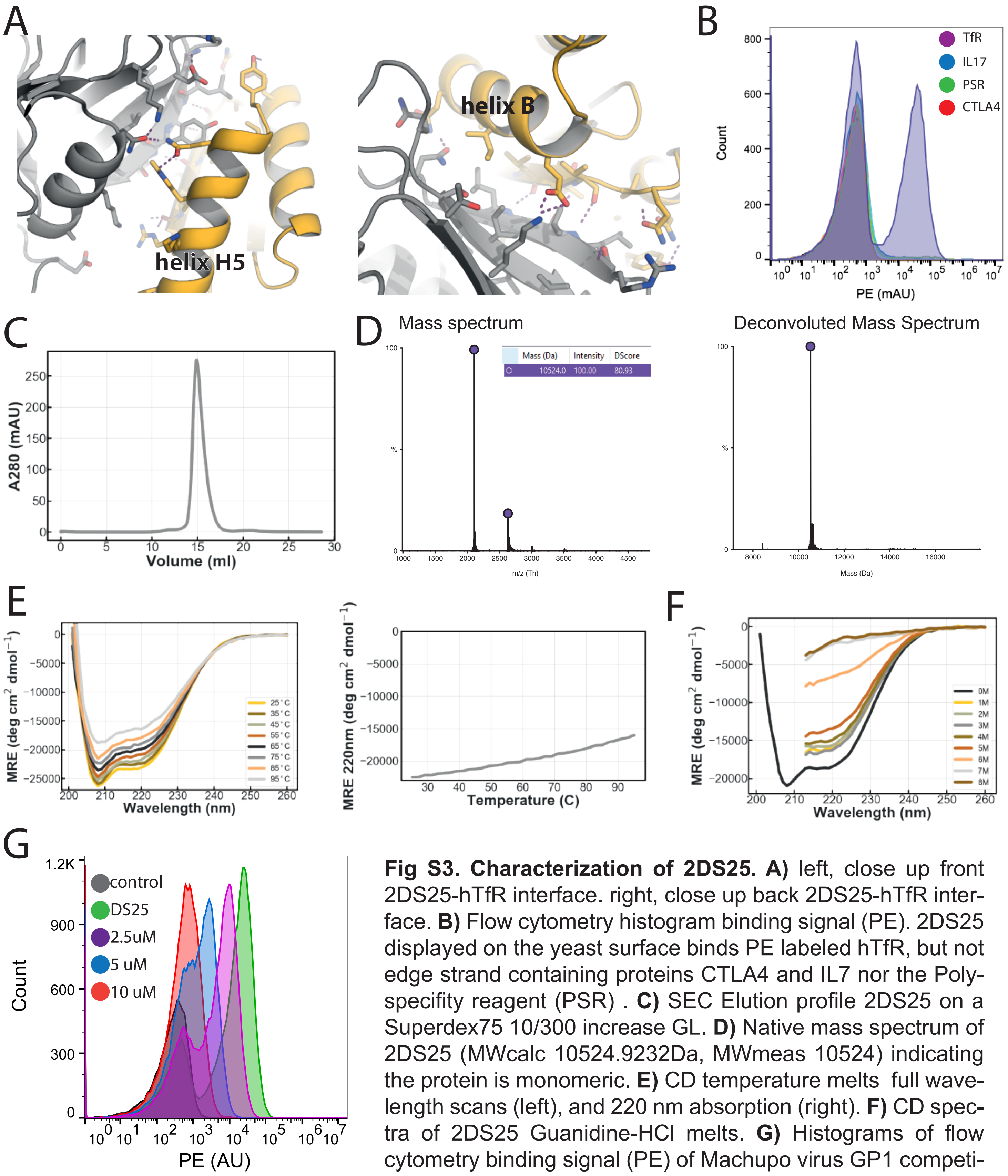

Fig S3. Characterization of 2DS25. A) left, close up front 2DS25-hTfR interface. right, close up back 2DS25-hTfR interface. **B)** Flow cytometry histogram binding signal (PE). 2DS25 displayed on the yeast surface binds PE labeled hTfR, but not edge strand containing proteins CTLA4 and IL7 nor the Polyspecifity reagent (PSR) . **C)** SEC Elution profile 2DS25 on a Superdex75 10/300 increase GL. **D)** Native mass spectrum of 2DS25 (MWcalc 10524.9232Da, MWmeas 10524) indicating the protein is monomeric. **E)** CD temperature melts full wavelength scans (left), and 220 nm absorption (right). **F)** CD spectra of 2DS25 Guanidine-HCl melts. **G)** Histograms of flow cytometry binding signal (PE) of Machupo virus GP1 competition experiments. 2DS25 on the yeast surface binds PE labeled hTfR (green). Addition of either 10,5 or 2.5 μM of Machupo GP1, which is known to bind the Apical domain of hTfR, reduces the binding signal in a dose dependent manner suggesting 2DS25 bind to its intended target region

Supplementary Figure 4

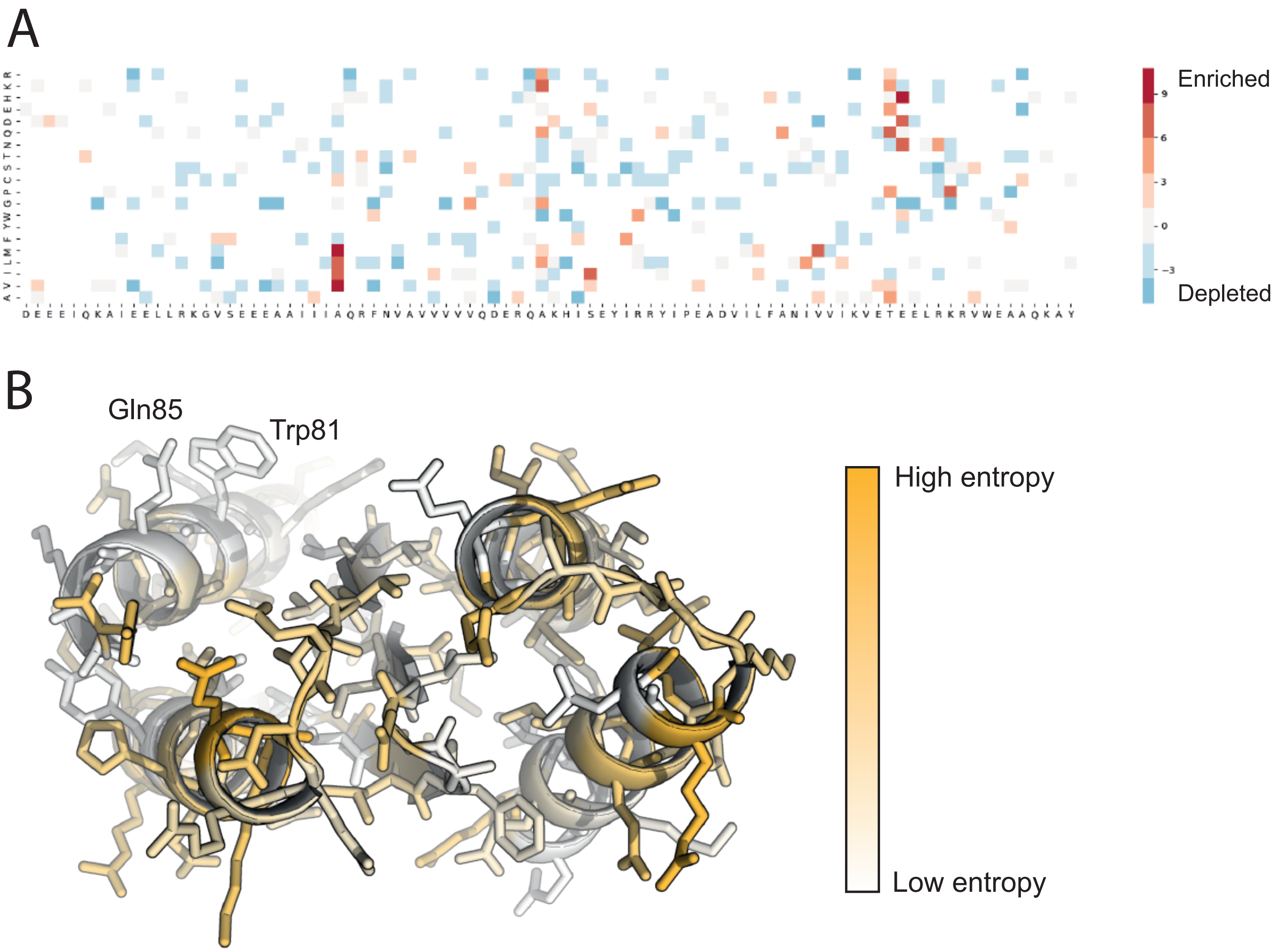

Fig S4. Site saturation mutagenesis (SSM) 2DS25. A) Representative heatmap (1 nM hTfR FACS sort) showing the enrichment values for each amino acid on each position. For mutants in white there was no data. **B)** 2DS25 colored by shannon entropy. Core residues in general have lower entropy than surface residues indicating that the design is correctly folded. Designed interface residues such as Trp81 and Gln85 have low entropy indicating these positions have low variability due to their importance for binding. The higher variability of several other interface residues was exploited to generate combination mutants in order to increase the affinity.

Supplementary Figure 5

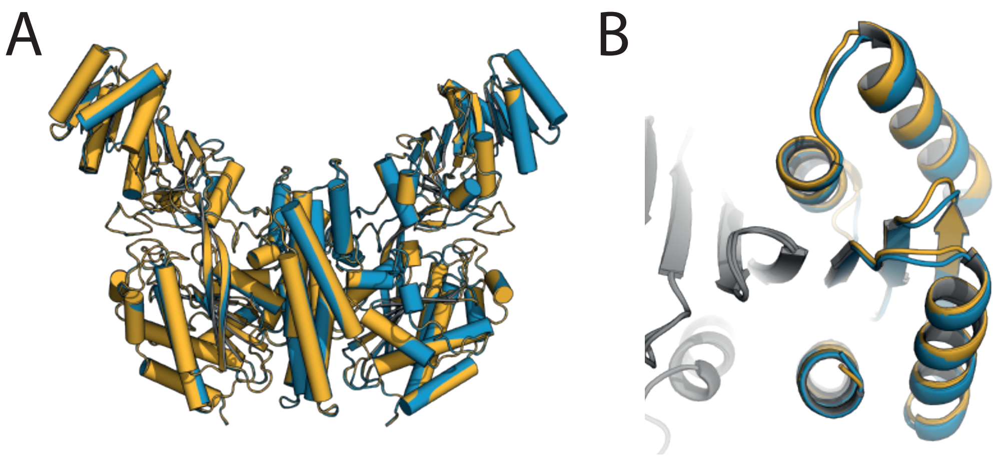

Fig S5. Crystal structure 2DS25.1 and 2DS25.5. A) Overview superposition of crystal structures of hTfR in complex with 2DS25.1 (blue) and 2DS25.5 (yellow) shows both structures are virtually identical. **B)** Closer view 2DS25 scaffolds.

Supplementary Figure 6

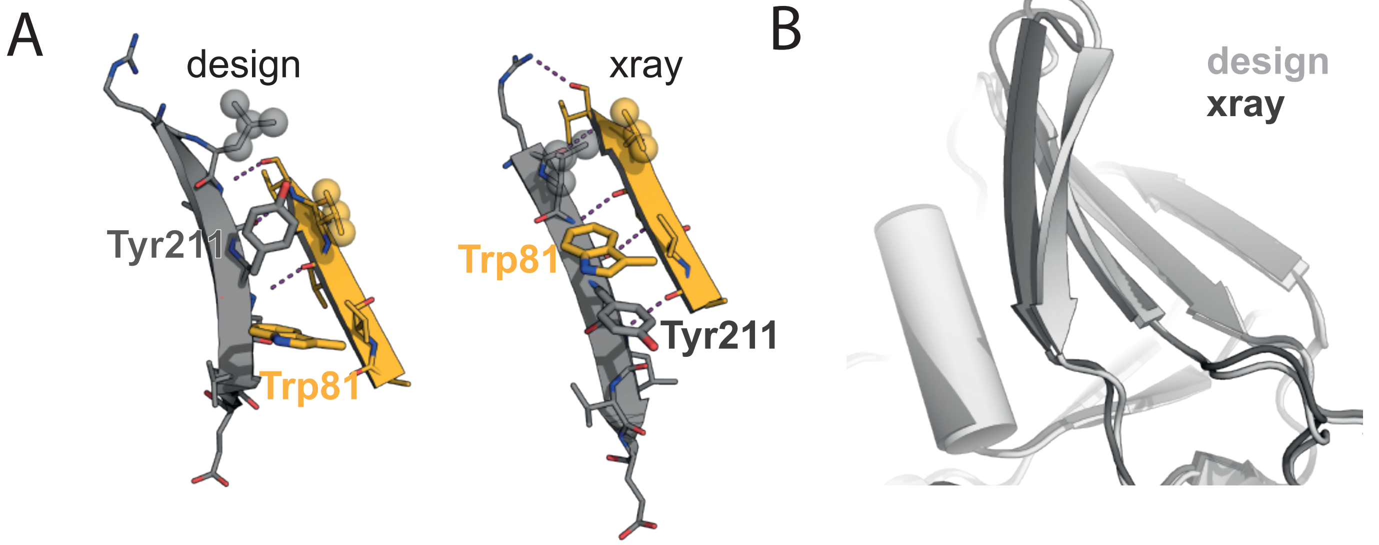

Fig S6. Strand shifting and conformational changes hTfR. A) Compared to the designed model left), a shift is observed in both the rotamer of Tyr211 and the beta sheet extension register in the crystal structure (right**). B)** The conformation of the hTfR edge strand in our crystal structures (dark gray) differs from the conformation in the structure used for design calculations (light gray, pdb code: 3kas).

Supplementary Figure 7

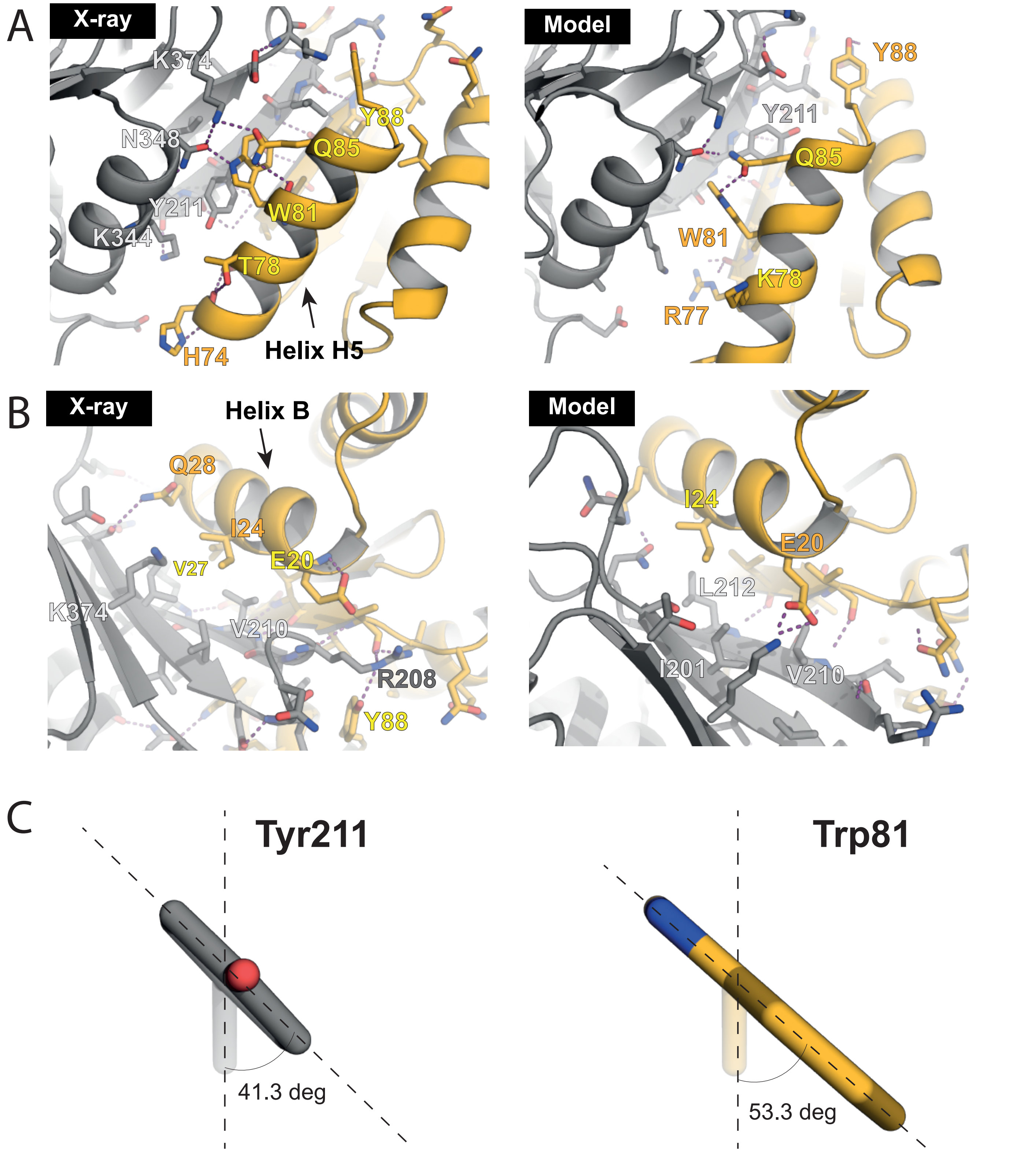

Fig S7. Details of the 2DS25-hTfR interface. A) Close up of the "front" interface of the 2DS25 model (right) and crystal structure (left) showing that in the crystal structure Trp81 makes a stacking interaction with Tyr211. **B)** Close up of the "back" interface featuring helix B of design strategy 2. 2DS25 model (right) and crystal structure (left) **C)** In the crystal structures both Tyr211 and Trp81 adopt strained χ_2 dihedral angles (angles from 2DS25.5/hTfR structure).

Supplementary Figure 8

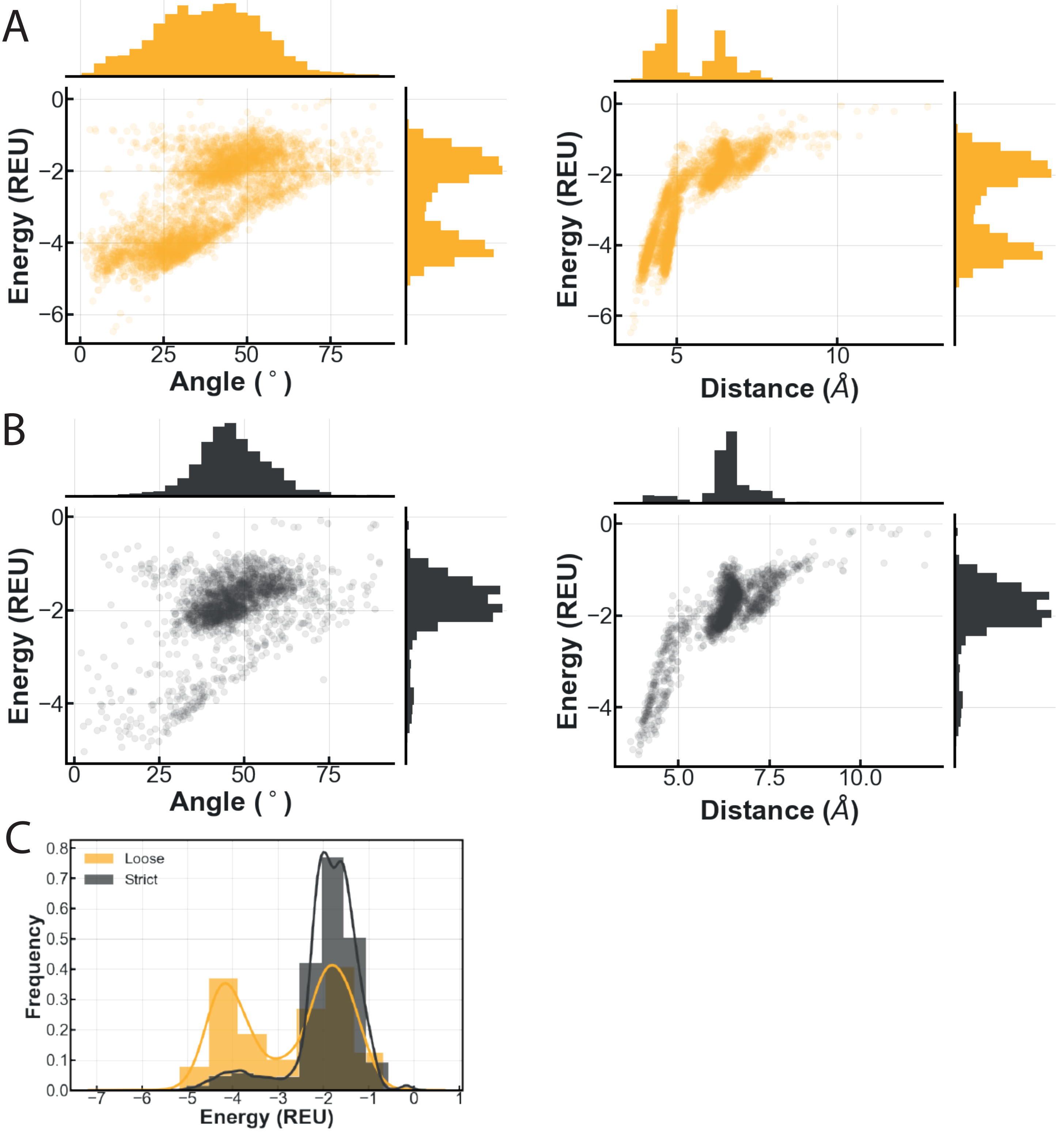

Fig S8. Altering chi2 constraints of aromatic residues promotes stacking interactions. Loosening the chi2 torsion angle restriction during design of the new docks from 70-110 degrees to 135-45 degrees favors face to face aromatic pi-pi stacking interactions to hTfR Tyr211. After sequence design we extracted all aromatic residues within interaction distance of Tyr211 for design runs with loosed chi2 **A)** and strict chi2 constraints **B)**. Scatterplots show relation between interaction energy and the angle between the planes of the two aromatic rings (typically <= 30 degrees for pi-pi stacks) and the relation of energy and the distance between the plane centroids of the aromatic rings (typically < 5 angstrom for pi-pi stacks). The low energy interactions show a clear dependence of energy on angle and distance indicating pi-pi stacking interactions. The number of favorable stacking interactions was much lower when the chi2 of aromatic residues was restricted to 70-110 degrees. **C)** When constraints were loosened, the percentage of designs with favorable interactions to Tyr211 increased dramatically from 0.85% (74/8676) to 15.3% (1329/8663)

Supplementary Figure 9

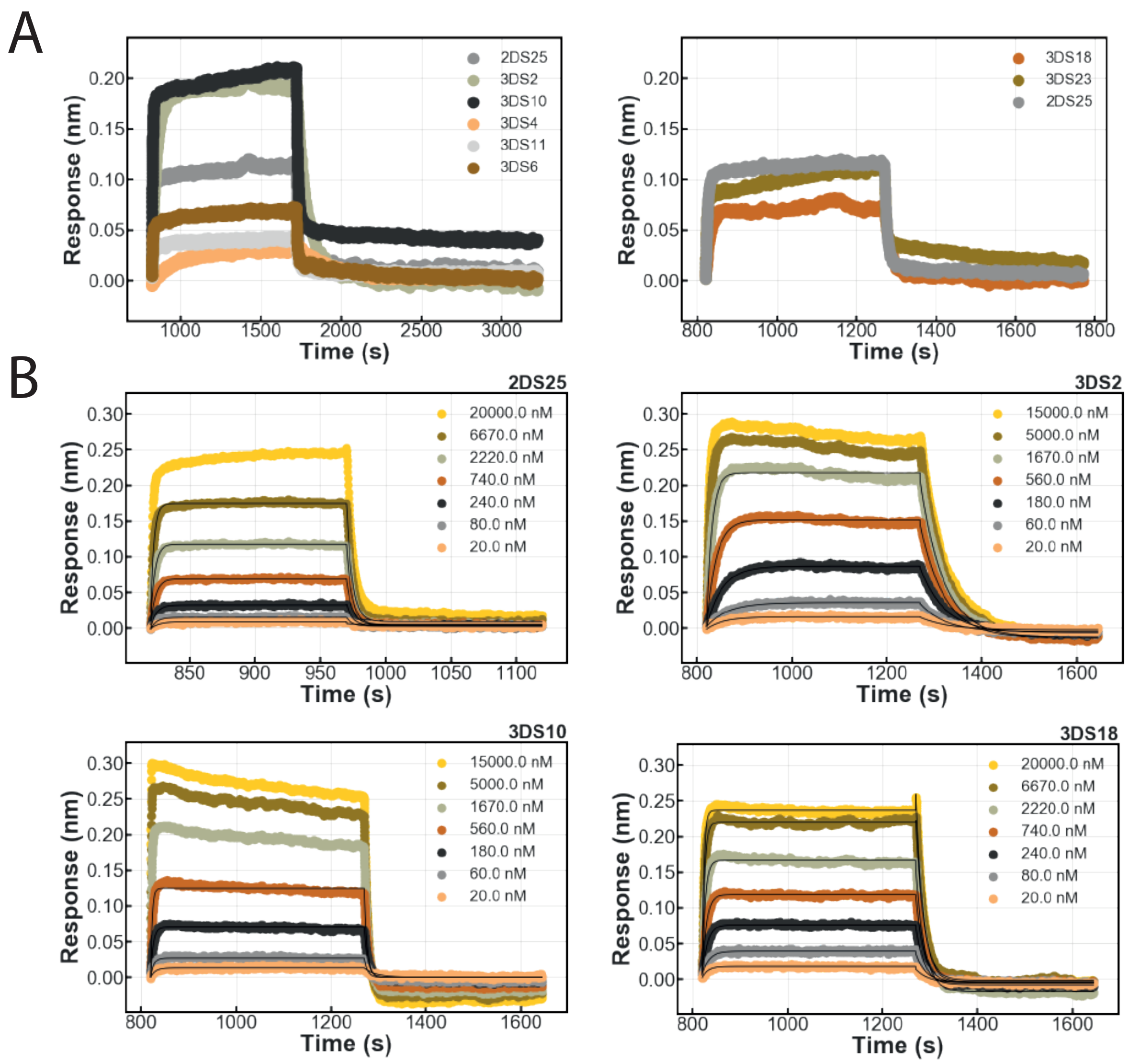

Fig S9. Biolayer interferometry traces third generation designs. A) Single concentration (1 µM) binding traces of 7 new designs binding to hTfR. **B)** Raw kinetic traces of designs 2DS25, 3DS2, 3DS10 and 3DS18 fitted to 1:1 binding model. Saturation binding curves of figure 3 were obtained from these data.

Supplementary Figure 10

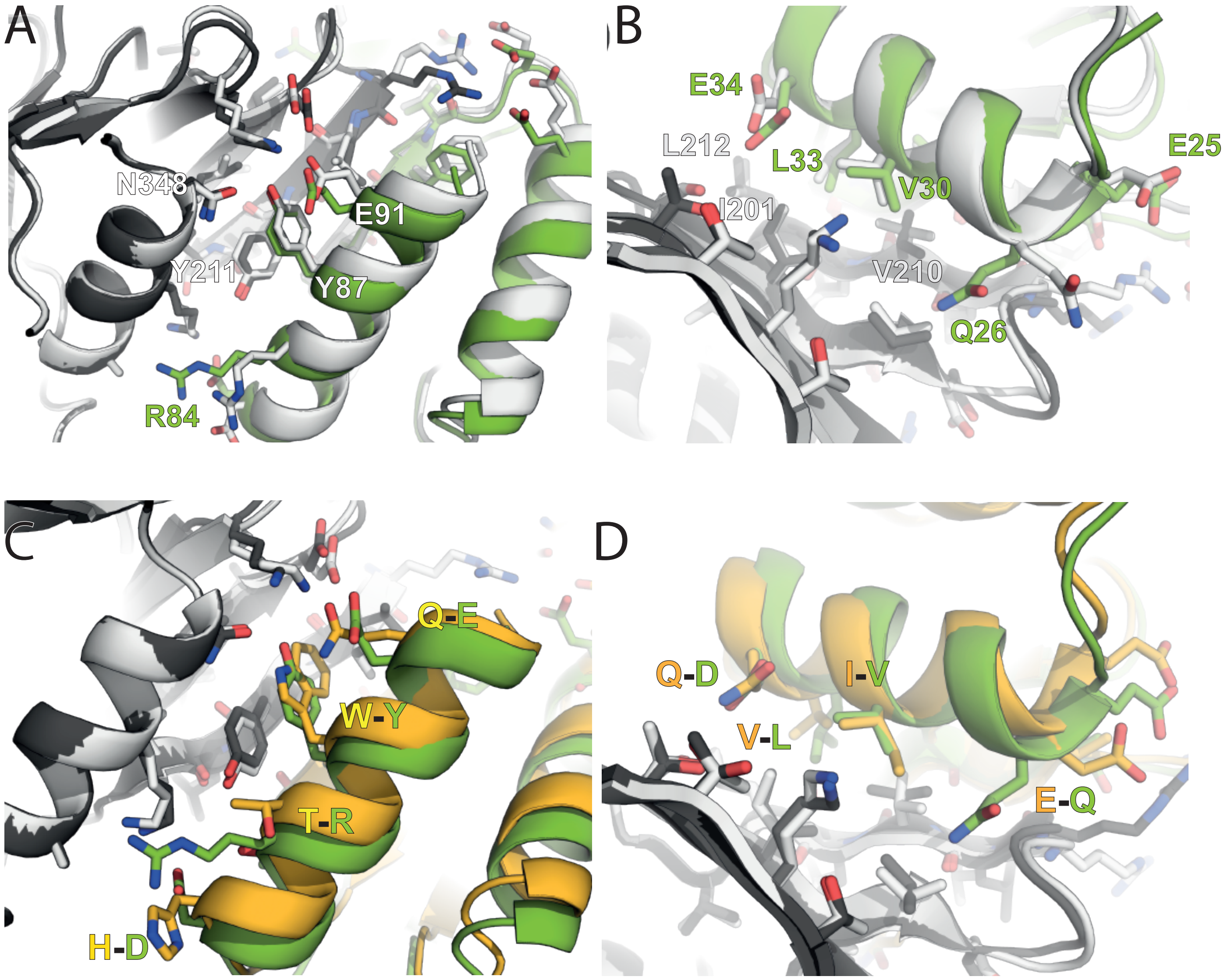

Fig S10. Details of the 3DS18-hTfR interface. A) Front view of the 3DS18-hTfR crystal structure (design in green and hTfR in dark gray) superimposed onto the designed model (light gray). **B)** Back view of the 3DS18-hTfR crystal structure (green and dark gray) superimposed onto the designed model (light gray). Overlay of the crystal structures of 3DS18 (green, light gray) and 2DS25.5 (yellow, dark gray) in complex with hTfR showing the front view **C)** and back view of the interface **D)**. Interface residues are shown in sticks.

Supplementary Figure 11

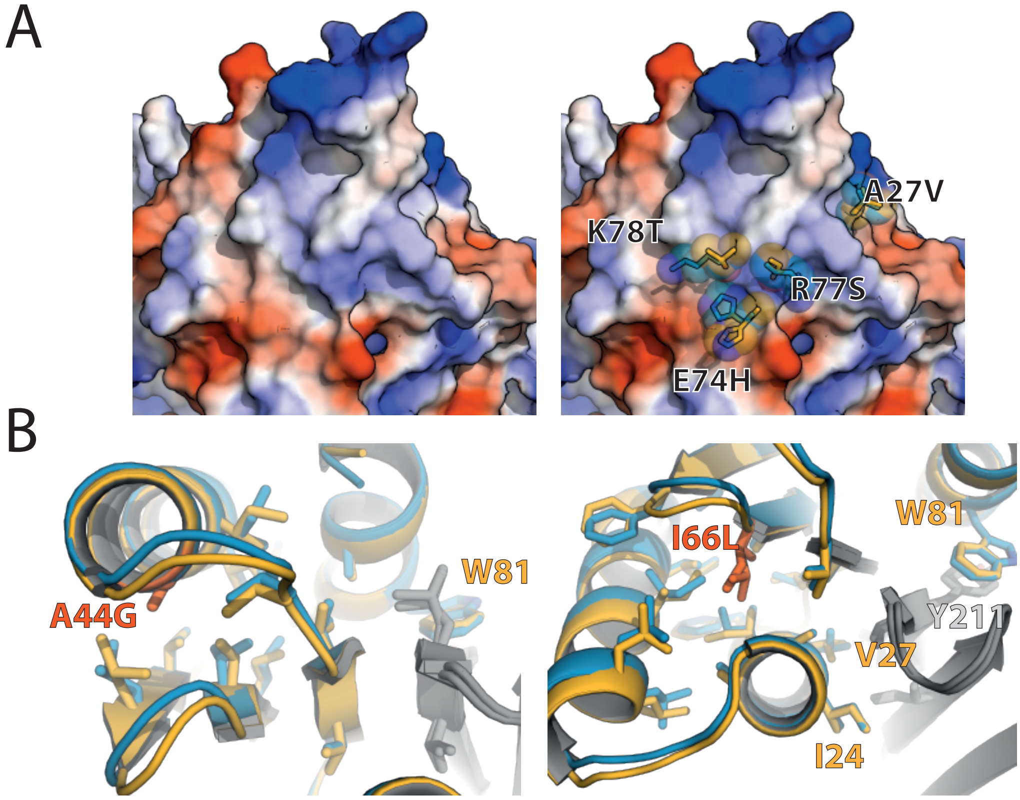

Fig S11. Optimization of the 2DS25 binder. A) Vacuum electrostatic potential surface map of hTfR generated in PyMOL suggests that the R77S and K78T substitutions may optimize binding by improving electrostatic complementarity to the target. **B)** Superposition of the crystal structure of 2DS25.5 (yellow) and 2DS25.1 (blue). Left, the A44G (orange) substitution identified in the SSM assists in improving binding to hTfR even though it is distal from the interface. Right, I66L has a similar effect as A44G despite not directly contacting hTfR. Both A44G and I66L are present in 2DS25.5 but not in 2DS25.1.

Supplementary figure 12

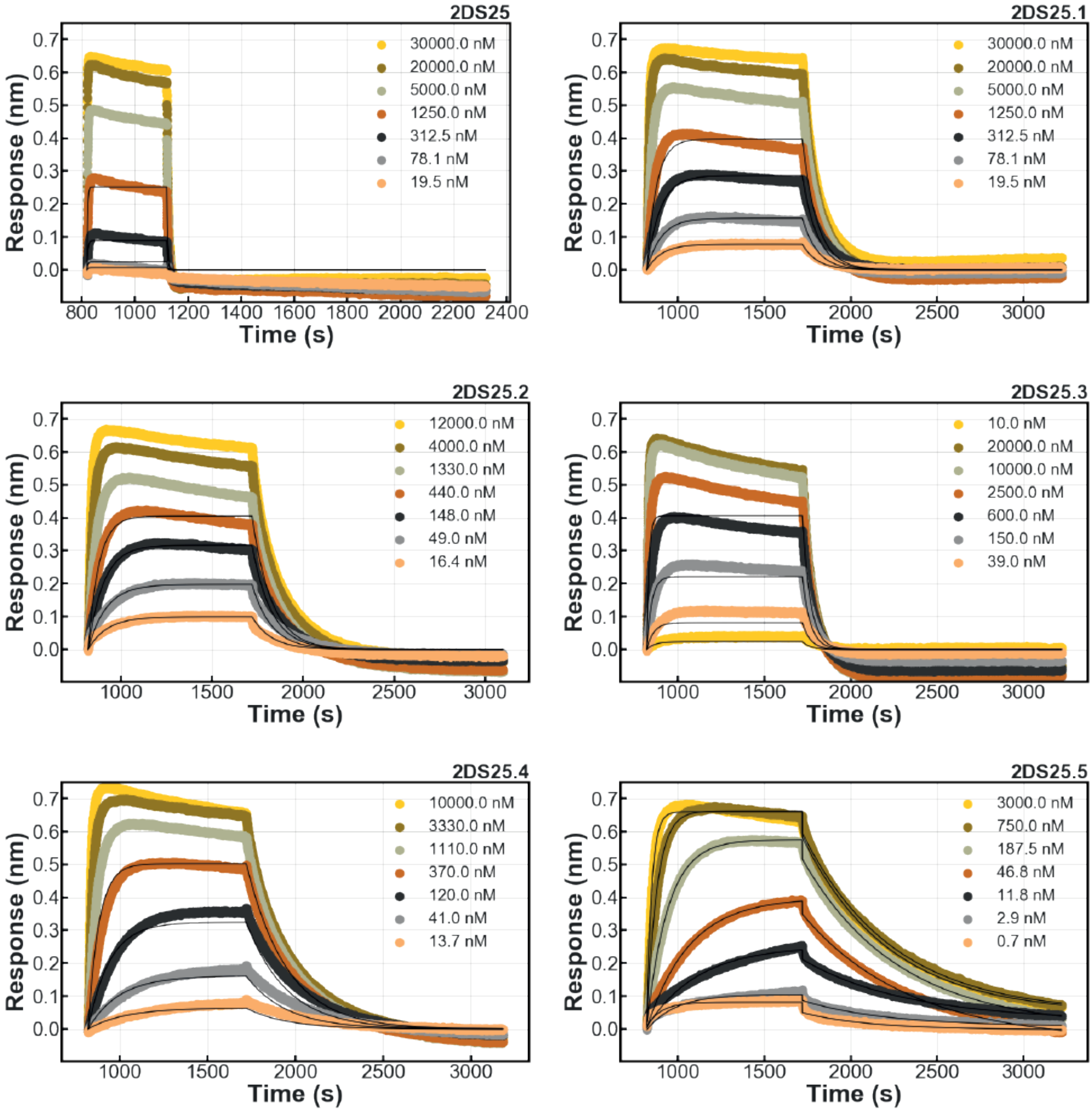

Fig S12. 2DS25 variants biolayer interferometry. Raw kinetic traces of 2DS25 variants binding to hTfR in biolayer interferometry binding experiments fitted to 1:1 binding model. Saturation binding curves from main figure 4 were obtained from these data.

Supplementary figure 13

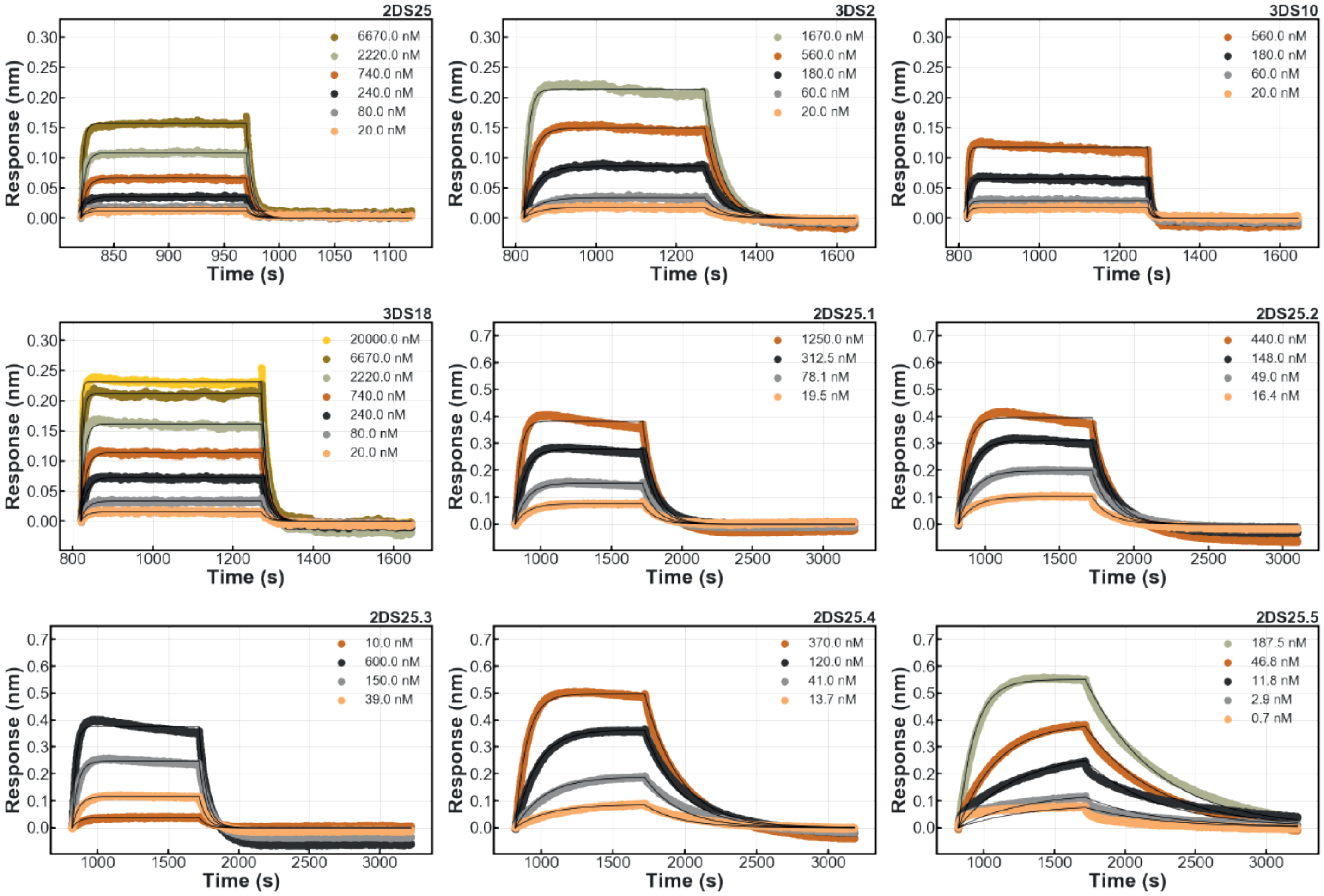

Fig S13. Global kinetic fits. Raw kinetic traces of designed variants binding to hTfR in biolayer interferometry binding with global kinetic fits. Global kinetic fitting was performed on the data from figure s9 and s12 to obtain Kd. Higher concentrations where binding response deviated from ideal behaviour were excluded. Kd's obtained from global kinetic fits and equilibrium response fits were similar (table s2).

Supplementary Tables

Table S1. Crystallographic data collection and refinement.

	**2DS25.1-hTfR_ECD (PDB: 6WRX)**	**2DS25.5-hTfR_ECD (PDB: 6WRW)**	**3DS18-hTfR_ECD (PDB: 6WRV)**
**Data Collection**			
Space group	$P\ 3_2\ 21$	$P\ 3_2\ 21$	$P\ 4_2\ 2_1\ 2$
Cell dimensions			
a, *b*, *c* (Å)	137.4, 137.4, 280.2	138.4, 138.4, 279.8	256.1,256.1, 128.1
α, *β*, *γ* (°)	90, 90, 120	90.0, 90.0, 120.0	90, 90, 90
Resolution (Å)	200.00-3.07 (3.25-3.07)	200.00-2.77 (2.94-2.77)	128.04-2.47 (2.51-2.47)
R_{merge} (%)	13.1 (156.4)	8.7 (147.0)	13.8(330.6)
R_{pim} (%)	6.2(98.7)	4.7(74.1)	3.7(86.7)
$I/\sigma(I)$	9.6 (1.03)	11.38 (1.01)	19.8 (0.9)
$CC_{1/2}$	0.997(0.404)	0.998 (0.338)	0.999(0.351)
Completeness (%)	99.8 (99.5)	97.7 (97.8)	100.0(100.0)
Redundancy	5.4 (5.4)	4.03 (4.07)	13.8 (14.5)
**Refinement**			
Resolution (Å)	93.39 - 3.07 (3.17-3.07)	69.20 - 2.84 (2.94-2.84)	128.04 - 2.47 (2.51- 2.47)
No. reflections	57506	72244	151526
R_{work} / R_{free} (%)	19/23 (33/35)	21/23 (38/40)	22/23 (41/44)
No. atoms	11753	11757	17756
Protein	11572	11544	17416
Carbohydrate	14	16	26
Ligand (Ca)	2	2	3
Water	n/a	n/a	n/a
Ramachandran Favored/allowed Outlier (%)	96.82/3.18	97.03/2.90	97.39/2.52
R.m.s. deviations			
Bond lengths (Å)	0.002	0.002	0.002
Bond angles (°)	0.45	0.43	0.51

$B_{factors}$ (Å^2)			
Protein	113.47	95.7	78.7
Carbohydrate	141.5	135.4	119.79
Ligand (Ca)	95.0	61.18	32.40
Water	n/a	n/a	n/a

Data were collected from one crystal per condition. [a] Values given in parentheses refer to reflections in the outer resolution shell. [b] $R_{sym} = \Sigma \mid I(k) - \langle I \rangle \mid / \Sigma I(k)$, where I(k) and <I> represent the scaled intensity values of the individual measurements and the corresponding mean values. For calculation of R_{free}, 5% of all reflections were omitted from refinement.

Table S2. Best fit Kd values from equilibrium and kinetic fits. Kinetic binding data was collected in a biolayer interferometry experiment (Octet, Forte bio). Data was processed and analyzed using octet data analysis software 9.1. Fitted values and their uncertainty sd are given.

**Design**		**Steady-state fit**			**Kinetic fit**		
**ID**	**Mutation**	**K_D (μM)**	**B_{MAX} (nm)**	**R-sqr**	**K_D (μM)**	**Chi-sqr**	**R-sqr**
2DS25	n/a	2.03 ± 3.1E-03	0.66 ± 0.02E-03	0.99	4.5 ± 0.25	0.025	0.99
2DS25.1	A27V/E74H	0.39 ± 0.12	0.61 ± 0.03	0.95	0.8 ± 0.14	1.3	0.99
2DS25.2	A27V/I66L/E74H	0.13 ± 0.032	0.59 ± 0.029	0.96	0.39 ± 0.12	4.17	0.98
2DS25.3	A27V/R29T/I66L/E74D/R77N	0.23 ± 0.05E-03	0.56 ± 0.02E-03	0.99	0.58 ± 0.35	6.8	0.96
2DS25.4	A27V/A44G/I66L/E74H	0.13 ± 0.02E-03	0.68 ± 0.02E-03	0.99	0.18 ± 0.02	1.34	0.99
2DS25.5	A27V/A44G/I66L/E74H/R77S/K78T	0.02 ± 0.004	0.64 ± 0.024	0.98	0.05 ± 0.004	2.9	0.99
3DS2	n/a	0.41 ± 0.022	0.27 ± 0.003	0.99	0.65 ± 0.012	0.11	0.99
3DS10	n/a	0.64 ± 0.052	0.28 ± 0.006	0.99	0.6 ± 0.06	0.08	0.98
3DS18	n/a	0.72 ± 0.14	0.23 ± 0.01	0.98	3.5 ± 0.09	0.2	0.99

Scripts and command line examples

The instructions found below should be viewed as a guideline. For more details on Rosetta scripts see https://www.rosettacommons.org/docs/latest/scripting_documentation/RosettaScripts/RosettaScripts

Example edge strand docking

The edge strand docking protocol makes use of PyRosetta 4, python 3.6 and several python packages. See code for more details.

Minimize target protein into Rosetta scorefunction using Rosetta FastRelax

```
python <path to dock_strands.py> -s <target pdb> -fastrelax
```

Performs kinematic (torsion) minimization with coordinate constraints (default 1.0). Change constraints by passing "-coordev" followed by float. The lower the value the stronger the constraints. Output will be renumbered to start at 1. This is important for many Rosetta pipelines.

Dock strand to user specified edge strand

After relaxing and renumbering (target numbering starts at 1), identify the target edge strand (in this example residue 12 to 16) and run the following command:

```
python <path to dock_strands.py> -s <target pdb> -edge 12-16 -database <path to betamotifs>
```

The database can be downloaded as a tarball from the online zenodo repository (DOI: 10.5281/zenodo.4594115). After untarring, the "lib" directory path should be passed as argument for -database

Dock strand to exposed edge strands (automatic)

After relaxing and renumbering (target numbering starts at 1), run the following command:

```
python <path to dock_strands.py> -s <target pdb> -autodetect -database <path to betamotifs>
```

The flag "-bb_only" can be added to perform the edge strand docking on a poly alanine target. This can be useful if there are side chains on the target that protect the edge strand. If there is reason to believe the side chain can move to deprotect the edge strand "-bb_only" is recommended

Minimize docked strands

python <path to minimize_strands.py> -s <target pdb> -strand <docked strand pdb> -threshold -4

Docked strands start with "aln_" prefix. Threshold energy can be increased or decreased. Lower thresholds will give docks with low energy (more bb hbonds across the beta sheet extension) but less output because it is more stringent. By default the target side chains are allowed to repack during the minimization. This can be prevented by adding the "-fix_target" flag. Alternatively if you only want certain residues on the target to repack you can pass the "-repack_target_residues" flag followed by a comma separated string of residues i.e. "-repack_target_residues 34,101".

Match dock strands to scaffold library

<path to rosetta>/main/source/bin/rosetta_scripts.default.linuxgccrelease -l <path to input list containing full paths scaffolds> -parser:protocol <path to design rosettascripts xml> -beta -parser:script_vars target=<path to target pdb> motif=<path to minimized strand>

The supporting_data.tar.gz contains a directory with example scaffolds used in this study. The data can be downloaded from the online zenodo repository (DOI: 10.5281/zenodo.4594115)

```
<ROSETTASCRIPTS>
    <MOVERS>
        <MotifGraft name="motifgraft" context_structure="%%target%%"
motif_structure="%%motif%%" RMSD_tolerance="0.5"
            NC_points_RMSD_tolerance="0.5" clash_score_cutoff="5" clash_test_residue="ALA"
combinatory_fragment_size_delta="2:2"
            max_fragment_replacement_size_delta="-1:1"  full_motif_bb_alignment="1"
allow_independent_alignment_per_fragment="0"
            graft_only_hotspots_by_replacement="0"
only_allow_if_N_point_match_aa_identity="0"
            only_allow_if_C_point_match_aa_identity="0" revert_graft_to_native_sequence="0"
allow_repeat_same_graft_output="1"/>
        <MultiplePoseMover name="mpm" max_input_poses="3">
            <ROSETTASCRIPTS>
                <SCOREFXNS>
                    <ScoreFunction name="NOV16_cart" weights="beta_cart" />
                    <ScoreFunction name="NOV16" weights="beta" >
                        <Reweight scoretype="fa_elec" weight="1.2" />
                        <Reweight scoretype="aa_composition" weight="1.0" />
                    </ScoreFunction>
                    <ScoreFunction name="VDW" weights="empty" >
                        <Reweight scoretype="fa_atr" weight="1.0" />
                    </ScoreFunction>
                </SCOREFXNS>

                <RESIDUE_SELECTORS>
                    <Chain name="chainA" chains="A"/>
                    <Chain name="chainB" chains="B"/>
                    <Neighborhood name="interface_chA" selector="chainB" distance="8.0"/>
                    <Neighborhood name="interface_chB" selector="chainA" distance="8.0"/>
                    <And name="AB_interface" selectors="interface_chA,interface_chB" />
```

```
                    <Not name="Not_interface" selector="AB_interface" />
                </RESIDUE_SELECTORS>

                <TASKOPERATIONS>
                    Graft
                    Design
                    <ProteinInterfaceDesign name="pack_long" design_chain1="0"
design_chain2="0" jump="1" interface_distance_cutoff="15"/>
                    <InitializeFromCommandline name="init"/>
                    <IncludeCurrent name="current"/>
                    <LimitAromaChi2 name="limitchi2" chi2max="135" chi2min="45"
include_trp="True" />
                    <ExtraRotamersGeneric name="ex1_ex2" ex1="1" ex2aro="1"/>
                    <ExtraRotamersGeneric name="ex1" ex1="1"/>
                    <LayerDesign name="all_layers"
layer="core_boundary_surface_Nterm_Cterm" use_sidechain_neighbors="True" pore_radius="2.0"
verbose="true" />
                    <OperateOnResidueSubset name="restrict_to_interface"
selector="Not_interface">
                        <PreventRepackingRLT/>
                    </OperateOnResidueSubset>
                    <OperateOnResidueSubset name="fix_target" selector="chainB">
                        <PreventRepackingRLT/>
                    </OperateOnResidueSubset>
                </TASKOPERATIONS>

                <MOVERS>
                    Graft
                    <MinMover name="kin_min" scorefxn="NOV16" chi="1" bb="1" cartesian="0"
type="dfpmin_armijo_nonmonotone" tolerance="0.01" max_iter="200">
                        <MoveMap name="MM" >
                            <Chain number="1" chi="true" bb="true"/>
                            <Chain number="2" chi="false" bb="false"/>
                            <Jump number="1" setting="false"/>
                        </MoveMap>
                    </MinMover>
                    <MinMover name="cart_min" scorefxn="NOV16_cart" chi="1" bb="1"
cartesian="1" omega="1" bondangle="0" bondlength="0" type="lbfgs_armijo_nonmonotone"
tolerance="0.01" max_iter="200">
                        <MoveMap name="MM" >
                            <Chain number="1" chi="true" bb="true"/>
                            <Chain number="2" chi="false" bb="false"/>
                            <Jump number="1" setting="false"/>
                        </MoveMap>
                    </MinMover>
                    Design
                    <!--Penalize excess tryp -->
                    <AddCompositionConstraintMover name="5trp" >
                        <Comp entry="PENALTY_DEFINITION;TYPE TRP;ABSOLUTE 0;PENALTIES 0
5;DELTA_START 0;DELTA_END 1;BEFORE_FUNCTION CONSTANT;AFTER_FUNCTION
LINEAR;END_PENALTY_DEFINITION;" />
                    </AddCompositionConstraintMover>
                    <AddHelixSequenceConstraints name="helix_seq_comp" />
                                <AtomTree name="docking_foldtree" docking_ft="true" />
                    <TaskAwareMinMover name="min" scorefxn="NOV16" bb="0" chi="1"
task_operations="pack_long"/>
                    <SwitchChainOrder name="switch_motifgraft_output" chain_order="21"/>
                    <SwitchChainOrder name="chain1only" chain_order="1"/>
                    <SwitchChainOrder name="chain2only" chain_order="2"/>
                    <Dssp name="dssp" reduced_IG_as_L="1"/>
                    <VirtualRoot name="add_virtual_root" removable="true" remove="false" />
```

```
                    <VirtualRoot name="remove_virtual_root" removable="true" remove="true"
/>
                    <PackRotamersMover name="quick_pack" scorefxn="NOV16"
task_operations="init,current,restrict_to_interface,fix_target" />
                    <FastDesign name="FastDesign" scorefxn="NOV16" repeats="2"
task_operations="init,current,limitchi2,all_layers,fix_target,restrict_to_interface,ex1"
                      batch="false" ramp_down_constraints="false"
                      cartesian="false" bondangle="false" bondlength="false"
                      min_type="dfpmin_armijo_nonmonotone" >
                        <MoveMap name="MM" >
                            <Chain number="1" chi="true" bb="true"/>
                            <Chain number="2" chi="false" bb="false"/>
                            <Jump number="1" setting="true"/>
                        </MoveMap>
                    </FastDesign>
                    <FastRelax name="FastRelax" scorefxn="NOV16" repeats="2" batch="false"
ramp_down_constraints="false" cartesian="false" bondangle="false" bondlength="false"
min_type="dfpmin_armijo_nonmonotone"
task_operations="init,current,limitchi2,ex1_ex2,fix_target" >
                        <MoveMap name="MM" >
                            <Chain number="1" chi="true" bb="true"/>
                            <Chain number="2" chi="false" bb="false"/>
                            <Jump number="1" setting="true"/>
                       </MoveMap>
                    </FastRelax>
                </MOVERS>

                <FILTERS>
                    <Geometry name="geometry_MBF" />
                    <MoveBeforeFilter name="geometry_monomer" mover="chain1only"
filter="geometry_MBF" confidence="1" />
                    <Sasa name="interface_buried_sasa" confidence="0"/>
                </FILTERS>

                <PROTOCOLS>
                    <Add mover_name="switch_motifgraft_output" />
                    <Add mover_name="quick_pack" />
                    <Add mover_name="cart_min" />
                    <Add mover_name="kin_min" />
                    <Add filter_name="geometry_monomer"/>
                    <Add filter_name="interface_buried_sasa"/>
                </PROTOCOLS>
            </ROSETTASCRIPTS>
        </MultiplePoseMover>
    </MOVERS>

    <PROTOCOLS>
        <Add mover_name="motifgraft"/>
        <Add mover_name="mpm"/>
    </PROTOCOLS>


</ROSETTASCRIPTS>
```

Running design RosettaScripts XML

<path to rosetta>/main/source/bin/rosetta_scripts.default.linuxgccrelease -s <path to input pdb> -parser:protocol <path to design rosettascripts xml> -beta

Scaffold design example xml

```
<ROSETTASCRIPTS>
    <SCOREFXNS>
        <ScoreFunction name="NOV16" weights="beta" >
            <Reweight scoretype="coordinate_constraint" weight="1" />
            <Reweight scoretype="aa_composition" weight="1.0" />
        </ScoreFunction>
    </SCOREFXNS>

    <RESIDUE_SELECTORS>
        <Chain name="chA" chains="A" />
    </RESIDUE_SELECTORS>

    <TASKOPERATIONS>
        <InitializeFromCommandline name="init"/>
        <IncludeCurrent name="current"/>
        <LimitAromaChi2 name="limitchi2" chi2max="110" chi2min="70" include_trp="True" />
        <ExtraRotamersGeneric name="ex1_ex2" ex1="1" ex2="1"/>
        <ExtraRotamersGeneric name="ex1" ex1="1"/>
        <DisallowIfNonnative name="disallow_GLY" resnum="0" disallow_aas="G" />
        <DisallowIfNonnative name="disallow_PRO" resnum="0" disallow_aas="P" />
        <LayerDesign name="all_layers" layer="core_boundary_surface_Nterm_Cterm"
use_sidechain_neighbors="True" pore_radius="2.0" verbose="true" />
    </TASKOPERATIONS>

    <MOVERS>
        <VirtualRoot name="add_virtual_root" removable="true" remove="false" />
        <VirtualRoot name="remove_virtual_root" removable="true" remove="true" />
        <AddHelixSequenceConstraints name="helix_seq_comp" />
        <AddCompositionConstraintMover name="alacomp" selector="chA">
            <Comp entry="PENALTY_DEFINITION;TYPE ALA;FRACT_DELTA_START
-0.03;FRACT_DELTA_END 0.03;PENALTIES 0 0 2;FRACTION 0.08;BEFORE_FUNCTION
CONSTANT;AFTER_FUNCTION QUADRATIC;END_PENALTY_DEFINITION" />
              </AddCompositionConstraintMover>
        <AddCompositionConstraintMover name="valcomp" selector="chA">
                      <Comp entry="PENALTY_DEFINITION;TYPE VAL;FRACT_DELTA_START
-0.03;FRACT_DELTA_END 0.03;PENALTIES 0 0 2;FRACTION 0.08;BEFORE_FUNCTION
CONSTANT;AFTER_FUNCTION QUADRATIC;END_PENALTY_DEFINITION" />
              </AddCompositionConstraintMover>
        <FastDesign name="FastDesign" scorefxn="NOV16" repeats="3"
task_operations="init,current,ex1_ex2,ex1,limitchi2,disallow_GLY,disallow_PRO,all_layers"
          batch="false" ramp_down_constraints="false"
          cartesian="false" bondangle="false" bondlength="false"
          min_type="dfpmin_armijo_nonmonotone" >
            <MoveMap name="MM" >
                <Chain number="1" chi="true" bb="true"/>
            </MoveMap>
        </FastDesign>

        <FastRelax name="FastRelax" scorefxn="NOV16" repeats="3" batch="false"
ramp_down_constraints="false" cartesian="false" bondangle="false" bondlength="false"
min_type="dfpmin_armijo_nonmonotone" task_operations="limitchi2" >
            <MoveMap name="MM" >
                <Chain number="1" chi="true" bb="true"/>
            </MoveMap>
        </FastRelax>
    </MOVERS>

    <FILTERS>
        <Geometry name="geometry" confidence="1" />
```

```
        <PackStat name="pack_stat" repeats="3" />
        <CavityVolume name="cavity_volume" />
        <SSShapeComplementarity name="ss_sc" helices="1" loops="1" />
        <ExposedHydrophobics name="exp_hphobics" />
        <BuriedUnsatHbonds2 name="unsat_hbond2" confidence="0" jump_number="0"/>
        <ResidueCount name="AlaCount" residue_types="ALA" max_residue_count="9999"
confidence="0"/>
        <ResidueCount name="ValCount" residue_types="VAL" max_residue_count="9999"
confidence="0"/>
    </FILTERS>

    <PROTOCOLS>
        <Add mover_name="helix_seq_comp" />
        <Add mover_name="alacomp" />
        <Add mover_name="valcomp" />
        <Add mover_name="FastDesign" />
        <Add mover_name="FastRelax" />
        <Add filter_name="pack_stat" />
        <Add filter_name="cavity_volume" />
        <Add filter_name="ss_sc" />
        <Add filter_name="exp_hphobics" />
        <Add filter_name="geometry" />
        <Add filter_name="unsat_hbond2" />
        <Add filter_name="AlaCount" />
        <Add filter_name="ValCount" />
    </PROTOCOLS>

</ROSETTASCRIPTS>
```

One sided interface design example

```
<ROSETTASCRIPTS>
    <SCOREFXNS>
        <ScoreFunction name="NOV16" weights="beta" >
            <Reweight scoretype="aa_composition" weight="1.0" />
        </ScoreFunction>
        <ScoreFunction name="VDW" weights="empty" >
            <Reweight scoretype="fa_atr" weight="1.0" />
        </ScoreFunction>
    </SCOREFXNS>

    <RESIDUE_SELECTORS>
        <Chain name="chainA" chains="A"/>
        <Chain name="chainB" chains="B"/>
        <Neighborhood name="interface_chA" selector="chainB" distance="8.0"/>
        <Neighborhood name="interface_chB" selector="chainA" distance="8.0"/>
        <And name="AB_interface" selectors="interface_chA,interface_chB" />
        <Not name="Not_interface" selector="AB_interface" />
    </RESIDUE_SELECTORS>

    <TASKOPERATIONS>
        <ProteinInterfaceDesign name="pack_long" design_chain1="0" design_chain2="0"
jump="1" interface_distance_cutoff="15"/>
        <InitializeFromCommandline name="init"/>
        <IncludeCurrent name="current"/>
        <LimitAromaChi2 name="limitchi2" chi2max="135" chi2min="45" include_trp="True" />
        <ExtraRotamersGeneric name="ex1_ex2" ex1="1" ex2="1"/>
        <ExtraRotamersGeneric name="ex1" ex1="1"/>
        <LayerDesign name="all_layers" layer="core_boundary_surface_Nterm_Cterm"
use_sidechain_neighbors="True" pore_radius="2.0" verbose="true" />
        <OperateOnResidueSubset name="restrict_to_interface" selector="Not_interface">
            <PreventRepackingRLT/>
```

```
        </OperateOnResidueSubset>
        <OperateOnResidueSubset name="restrict_target" selector="chainB">
            <PreventRepackingRLT/>
        </OperateOnResidueSubset>
        <DisallowIfNonnative name="disallow" disallow_aas="GC" />
    </TASKOPERATIONS>

    <MOVERS>
        <AddHelixSequenceConstraints name="helix_seq_comp" />
        <AddCompositionConstraintMover name="addcomp" >
            <Comp entry="PENALTY_DEFINITION;TYPE ALA;FRACT_DELTA_START
-0.03;FRACT_DELTA_END 0.075;PENALTIES 0 0 2;FRACTION 0.07;BEFORE_FUNCTION
CONSTANT;AFTER_FUNCTION QUADRATIC;END_PENALTY_DEFINITION"/>
        </AddCompositionConstraintMover>
        <AtomTree name="docking_foldtree" docking_ft="true" />
        <TaskAwareMinMover name="min" scorefxn="NOV16" bb="0" chi="1"
task_operations="pack_long"/>
        <SwitchChainOrder name="chain1only" chain_order="1"/>
        <SwitchChainOrder name="chain2only" chain_order="2"/>
        <Dssp name="dssp" reduced_IG_as_L="1"/>
        <VirtualRoot name="add_virtual_root" removable="true" remove="false" />
        <VirtualRoot name="remove_virtual_root" removable="true" remove="true" />
        <FastDesign name="FastDesign" scorefxn="NOV16" repeats="3"
task_operations="init,current,limitchi2,ex1_ex2,ex1,all_layers,restrict_to_interface,disall
ow,restrict_target"
          batch="false" ramp_down_constraints="false"
          cartesian="false" bondangle="false" bondlength="false"
          min_type="dfpmin_armijo_nonmonotone" >
            <MoveMap name="MM" >
                <Chain number="1" chi="true" bb="true"/>
                <Chain number="2" chi="false" bb="false"/>
                <Jump number="1" setting="true"/>
            </MoveMap>
        </FastDesign>
        <FastRelax name="FastRelax" scorefxn="NOV16" repeats="3" batch="false"
ramp_down_constraints="false" cartesian="false" bondangle="false" bondlength="false"
min_type="dfpmin_armijo_nonmonotone" task_operations="init,current,limitchi2" >
            <MoveMap name="MM" >
                <Chain number="1" chi="true" bb="true"/>
                <Chain number="2" chi="false" bb="false"/>
                <Jump number="1" setting="true"/>
           </MoveMap>
        </FastRelax>
    </MOVERS>

    <FILTERS>
        <Ddg name="ddg"  threshold="-10" jump="1" repeats="5" repack="1" relax_mover="min"
confidence="0" scorefxn="NOV16" />
        <Ddg name="ddg_norepack"  threshold="-10" jump="1" repeats="1" repack="0"
confidence="0" scorefxn="NOV16"/>
        <Ddg name="ddg_fa_atr"  threshold="-10" jump="1" repeats="5" repack="1"
relax_mover="min" confidence="0" scorefxn="VDW" />
        <Ddg name="ddg_fa_atr_norepack"  threshold="-10" jump="1" repeats="1" repack="0"
confidence="0" scorefxn="VDW"/>
        <Sasa name="interface_buried_sasa" confidence="0" />
        <Sasa name="interface_hydrophobic_sasa" confidence="0" hydrophobic="True"/>
        <Sasa name="interface_polar_sasa" confidence="0" polar="True"/>
        <ShapeComplementarity name="interface_sc" verbose="0" min_sc="0.60"
write_int_area="1" jump="1" confidence="0"/>
        <BuriedUnsatHbonds2 name="interface_unsat_hbond2" confidence="0" jump_number="1"/>
```

```
        <BuriedUnsatHbonds name="interface_unsat_hbonds_new"
report_all_heavy_atom_unsats="true"  scorefxn="NOV16" ignore_surface_res="true"
print_out_info_to_pdb="true" confidence="0" residue_selector="AB_interface"/>
        <CalculatorFilter name="ddg_per_1000sasa" equation="1000 * ddg / (sasa+0.01)"
threshold="-25" confidence="0">
            <Var name="ddg" filter="ddg"/>
            <Var name="sasa" filter="interface_buried_sasa"/>
        </CalculatorFilter>
        <CalculatorFilter name="ddg_norepack_per_1000sasa" equation="1000 * ddg /
(sasa+0.01)" threshold="1" confidence="0">
            <Var name="ddg" filter="ddg_norepack"/>
            <Var name="sasa" filter="interface_buried_sasa"/>
        </CalculatorFilter>
        <CalculatorFilter name="ddg_fa_atr_per_1000sasa" equation="1000 * ddg /
(sasa+0.01)" threshold="1" confidence="0">
            <Var name="ddg" filter="ddg_fa_atr"/>
            <Var name="sasa" filter="interface_buried_sasa"/>
        </CalculatorFilter>
        <CalculatorFilter name="ddg_fa_atr_norepack_per_1000sasa" equation="1000 * ddg /
(sasa+0.01)" threshold="1" confidence="0">
            <Var name="ddg" filter="ddg_fa_atr_norepack"/>
            <Var name="sasa" filter="interface_buried_sasa"/>
        </CalculatorFilter>
        <CalculatorFilter name="interface_fxn_hydrophobic" equation="hydrophobic / (sasa +
0.01)" threshold="1" confidence="0">
            <Var name="hydrophobic" filter="interface_hydrophobic_sasa"/>
            <Var name="sasa" filter="interface_buried_sasa"/>
        </CalculatorFilter>
        <ScoreType name="hbond_lr_bb_complex" scorefxn="NOV16" score_type="hbond_lr_bb"
threshold="0" confidence="0" />
        <ScoreType name="hbond_lr_bb_MBF" scorefxn="NOV16" score_type="hbond_lr_bb"
threshold="0" confidence="0" />
        <MoveBeforeFilter name="hbond_lr_bb_A" mover="chain1only" filter="hbond_lr_bb_MBF"
confidence="0" />
        <MoveBeforeFilter name="hbond_lr_bb_B" mover="chain2only" filter="hbond_lr_bb_MBF"
confidence="0" />
        <CalculatorFilter name="delta_hbond_lr_bb" equation="A + B - complex"
threshold="-2.0" confidence="0">
            <Var name="complex" filter="hbond_lr_bb_complex"/>
            <Var name="A" filter="hbond_lr_bb_A"/>
            <Var name="B" filter="hbond_lr_bb_B"/>
        </CalculatorFilter>
        <ScoreType name="total_score_MBF" scorefxn="NOV16" score_type="total_score"
threshold="0" confidence="0" />
        <MoveBeforeFilter name="total_score_monomerA" mover="chain1only"
filter="total_score_MBF" confidence="0" />
        <BuriedUnsatHbonds2 name="unsat_hbond2_monomer_MBF" confidence="0"
jump_number="0"/>
        <MoveBeforeFilter name="unsat_hbond2_monomerA" mover="chain1only"
filter="unsat_hbond2_monomer_MBF" />
        <ResidueCount name="count_MBF" />
        <MoveBeforeFilter name="countA" mover="chain1only" filter="count_MBF"/>
        <MoveBeforeFilter name="countB" mover="chain2only" filter="count_MBF"/>
        <CalculatorFilter name="score_per_resA" equation="total_score_monomer / res"
threshold="-2.5" confidence="0">
            <Var name="total_score_monomer" filter="total_score_monomerA"/>
            <Var name="res" filter="countA"/>
        </CalculatorFilter>
    </FILTERS>

    <PROTOCOLS>
        <Add mover_name="helix_seq_comp" />
```

```
        <Add mover_name="addcomp" />
        <Add mover_name="docking_foldtree" />
        <Add mover_name="FastDesign" />
        <Add mover_name="FastRelax" />
        <Add filter_name="ddg" />
        <Add filter_name="ddg_norepack"/>
        <Add filter_name="ddg_fa_atr" />
        <Add filter_name="ddg_fa_atr_norepack"/>
        <Add filter_name="interface_buried_sasa"/>
        <Add filter_name="interface_hydrophobic_sasa" />
        <Add filter_name="interface_polar_sasa" />
        <Add filter_name="interface_sc"/>
        <Add filter_name="interface_unsat_hbond2"/>
        <Add filter_name="interface_unsat_hbonds_new"/>
        <Add filter_name="ddg_per_1000sasa"/>
        <Add filter_name="ddg_norepack_per_1000sasa"/>
        <Add filter_name="ddg_fa_atr_per_1000sasa"/>
        <Add filter_name="ddg_fa_atr_norepack_per_1000sasa"/>
        <Add filter_name="interface_fxn_hydrophobic" />
        <Add filter_name="total_score_monomerA" />
        <Add filter_name="unsat_hbond2_monomerA"/>
        <Add filter_name="score_per_resA" />
        <Add filter_name="hbond_lr_bb_complex" />
        <Add filter_name="delta_hbond_lr_bb" />
    </PROTOCOLS>
</ROSETTASCRIPTS>
```

Example backbone generation

Blueprint generation

<path to rosetta>/main/source/bin/make_blueprint.linuxgccrelease -s <path pdb of which to make blueprint>

Edit blueprint file

In a text editor edit the blueprint file to add loops/secondary structure elements. See https://www.rosettacommons.org/docs/latest/scripting_documentation/RosettaScripts/Movers/movers_pages/BluePrintBDRMover for details.

```
# Example blueprint file that adds a loop of "AGB" type and 13 residue helix to the
N-terminus # of an input pdb.
#### SS_Info
# Helix  1: 10-23
# Helix  2: 43-56
# Strand 1: 2-6
# Strand 2: 28-32
# Strand 3: 35-39
#### StrandPairingSet Info
# 1-3.A.0;2-3.A.0
# 1-3.A.0: 2-6.39-35
# 2-3.A.0: 28-32.39-35
SSPAIR 1-3.A.0;2-3.A.0
#### Sheet Info
## Sheet 1:
#Strand 1: 1,1
#Strand 2: 3,-1
#Strand 3: 2,1
```

```
### BetaAlphaBetaMotif Info
# helix,strand1-strand2.num_crossover hsheet_dist hs_angl hsheet_elev_angl hs1_dist
hs2_dist helix_cycle
# 1,1-2.1 8.65838 -5.39169 -15.1451 11.2617 8.8569 14
0 V HA R
0 V HA R
0 V HA R
0 V HA R
0 V HA R
0 V HA R
0 V HA R
0 V HA R
0 V HA R
0 V HA R
0 V HA R
0 V HA R
0 V HA R
0 V LA R
0 V LG R
1 A LB R
2 T EB .
3 I EB .
4 T EB .
5 V EB .
6 T EB .
7 A LB .
8 S LA .
9 N LB .
10 E HA .
11 E HA .
12 E HA .
13 A HA .
14 K HA .
15 K HA .
16 V HA .
17 A HA .
18 K HA .
19 I HA .
20 I HA .
21 H HA .
22 S HA .
23 L HA .
24 F LB .
25 P LA .
26 S LA .
27 V LB .
28 R EB .
29 C EB .
30 E EB .
31 T EB .
32 K EB .
33 D LG .
34 G LG .
35 T EB .
36 V EB .
37 Y EB .
38 I EB .
39 Y EB .
40 C LB .
41 Q LA .
42 T LB .
43 Q HA .
```

```
44 K HA .
45 Q HA .
46 A HA .
47 S HA .
48 E HA .
49 C HA .
50 L HA .
51 E HA .
52 V HA .
53 A HA .
54 H HA .
55 K HA .
56 A HA .
57 T LO .
```

Blueprint based backbone building

<path to rosetta>/main/source/bin/rosetta_scripts.default.linuxgccrelease -s <path to pdb that will be modified> -parser:protocol <path to design xml> -parser:script_vars blueprint=<path to blueprint file>

```
<ROSETTASCRIPTS>
<SCOREFXNS>
    <ScoreFunction name="CENTROID" weights="fldsgn_cen" symmetric="0" >
        <Reweight scoretype="hbond_lr_bb" weight="1.0" />
        <Reweight scoretype="hbond_sr_bb" weight="1.0" />
        <Reweight scoretype="atom_pair_constraint" weight="1.0" />
        <Reweight scoretype="coordinate_constraint" weight="1.0" />
        <Reweight scoretype="angle_constraint" weight="1.0" />
        <Reweight scoretype="dihedral_constraint" weight="1.0" />
        <Reweight scoretype="backbone_stub_constraint" weight="1.0" />
        <Reweight scoretype="omega" weight="0.5" />
        <Reweight scoretype="rama" weight="0.6" />
    </ScoreFunction>
</SCOREFXNS>

<FILTERS>
    <Geometry name="geometry" count_bad_residues="true" />
</FILTERS>

<TASKOPERATIONS>
</TASKOPERATIONS>

<MOVERS>
    <Dssp name="dssp" />
    <BluePrintBDR name="bdr1" use_abego_bias="1" scorefxn="CENTROID"
constraints_NtoC="1.0" blueprint="%%blueprint%%" />
    <DumpPdb name="dump" fname="%%blueprint%%_pass_" tag_time="True"/>
    <ParsedProtocol name="build_dssp1" >
        <Add mover_name="bdr1" />
        <Add mover_name="dssp" />
        <Add filter_name="geometry" />
        <Add mover_name="dump" />
    </ParsedProtocol>
    <LoopOver name="lover1" mover_name="build_dssp1" iterations="10" drift="0"
ms_whenfail="FAIL_DO_NOT_RETRY" />
    <ParsedProtocol name="phase1" >
        <Add mover_name="lover1" />
    </ParsedProtocol>
</MOVERS>
```

```
<PROTOCOLS>
    <Add mover_name="phase1" />
</PROTOCOLS>
</ROSETTASCRIPTS>
```

Edgestrand docking code

dock_strands.py

```
'''
Danny D. Sahtoe
dsahtoe@uw.edu, danny.sahtoe@gmail.com
University of Washington, Institute for Protein Design
Baker Lab
Created Aug 2016
'''
try:
    import sys
    import subprocess
    import argparse
    import glob
    import itertools
    import os
    from operator import itemgetter
    from itertools import groupby
    from Bio.PDB import *
    import pyrosetta
    import numpy as np
    from pprint import pprint
except ImportError:
    print('\nOne or more critical modules are not detected on your system.
Exiting.')
    sys.exit(1)

# Setup commandline options
parser = argparse.ArgumentParser()
parser.add_argument('-s', type=str, metavar='\b', default=None,
                    help='Specify input pdb', required=True)
parser.add_argument('-edge', type=str, metavar='\b', default=None,
                    help='Specify edgestrand range e.g. 12-14 to select resi
12,13,14. Needs to be at least 3 residues')
parser.add_argument('-database', type=str, metavar='\b', default=None,
required=False,
                    help='Specify full path to database containing beta
motifs')
parser.add_argument('-autodetect', action='store_true',
                    help='Autodetect edgestrands; cannot be used in
conjunction with -edge option')
#parser.add_argument('-autodetect_only', action='store_true',
```

```
#                     help='Only output pml script with autodetected
edgestrands highlighted')
parser.add_argument('-bb_only', action='store_true',
                    help='Ignore sidechains when detecting edgestrand for
example when flexible side chain covers otherwise accesible edge. Useful in
autodetect_mode')
parser.add_argument('-fastrelax', action='store_true',
                    help='Minimization at torsion level, NOT cartesian.')
parser.add_argument('-coordev', type=float, metavar='\b', default=None,
required=False,
                    help='Strength of constraints in fastrelax, the lower the
float the stronger the constraints. By default 1.0 if not set' )
args = parser.parse_args()


def fastrelax(pose,coordev):
    '''Kinematic minimization with Rosetta FastRelax. Min with coord csts'''
    #Setup fast_relax
    scorefxn = pyrosetta.get_fa_scorefxn()
    scorefxn(pose)
    fast_relax = pyrosetta.rosetta.protocols.relax.FastRelax(2)

scorefxn.set_weight(pyrosetta.rosetta.core.scoring.ScoreType.coordinate_const
raint,1.0)
    fast_relax.set_scorefxn(scorefxn)
    # setup coord cst
    addcsts = pyrosetta.rosetta.protocols.relax.AtomCoordinateCstMover()
    addcsts.cst_sd(coordev)
    addcsts.apply(pose)
    #setup task ops
    tf = pyrosetta.rosetta.core.pack.task.TaskFactory()

tf.push_back(pyrosetta.rosetta.core.pack.task.operation.InitializeFromCommand
line())
    tf.push_back(pyrosetta.rosetta.core.pack.task.operation.IncludeCurrent())

tf.push_back(pyrosetta.rosetta.core.pack.task.operation.NoRepackDisulfides())

tf.push_back(pyrosetta.rosetta.core.pack.task.operation.RestrictToRepacking()
)
    ex1_ex2 =
pyrosetta.rosetta.core.pack.task.operation.ExtraRotamersGeneric()
    ex1_ex2 =
pyrosetta.rosetta.core.pack.task.operation.ExtraRotamersGeneric()
    ex1_ex2.ex1(True)
    ex1_ex2.ex2(True)
    tf.push_back(ex1_ex2)
    limitchi2aro =
pyrosetta.rosetta.protocols.task_operations.LimitAromaChi2Operation()
    limitchi2aro.include_trp(True)
    limitchi2aro.chi2max(110)
```

```
    limitchi2aro.chi2min(70)
    tf.push_back(limitchi2aro)
    task = tf.create_task_and_apply_taskoperations(pose)
    #Setup common movemap elements i.e. jump=True and always alllow bb and
sidechain min.
    mm = pyrosetta.rosetta.core.kinematics.MoveMap()
    # Set jump to true if jump(s) present --> sets all jumps to True
    mm.set_chi(True)
    mm.set_bb(True)
    fast_relax.set_movemap(mm)
    fast_relax.set_task_factory(tf)
    fast_relax.apply(pose)
    print(('total_score',scorefxn(pose)))
    pyrosetta.dump_pdb(pose,'relax_renumbered_'+pdb.split('/')[-1])


def check_for_strands(pose):
    sheetsel =
pyrosetta.rosetta.core.select.residue_selector.SecondaryStructureSelector(
        'E')
    sheetsel.set_minE(3)  # minimum length of a strand
    betaresidues = pyrosetta.rosetta.core.select.get_residues_from_subset(
        sheetsel.apply(pose))
    return betaresidues


def switch_renumber(pose, pdbname):
    switch = pyrosetta.rosetta.protocols.simple_moves.SwitchChainOrderMover()
    switch.chain_order('1')
    switch.apply(pose)
    pyrosetta.dump_pdb(pose, pdbname)


def get_abego(betastretch):
    abegos = pyrosetta.rosetta.core.sequence.get_abego(pose, 1)
    abegostretch = []
    for i in betastretch:
        abegostretch.append(abegos[i])
    return abegostretch


def check_longest_beta(beta_stretch):
    long_enough = False
    group = [list(j) for i, j in groupby(beta_stretch)]
    for i in group:
        if 'B' in i and len(i) >= 4:
            long_enough = True
    return long_enough


def new_get_edgestrands(pose, pdbname):
```

```
    # Get all beta residues in a list
    betaresidues = check_for_strands(pose)
    # Make dict where you for each beta residue store per atom sasa of NH 'H'
and CO 'O'
    sasa = pyrosetta.rosetta.core.scoring.sasa.SasaCalc()
    sasa.calculate(pose)
    atomsasa = {}
    atoms = ['H', 'O']
    prolines = []
    for resn in betaresidues:
        resobj = pose.residue(resn)
        atomsasa[resn] = []
        if resobj.annotated_name() == 'P':
            prolines.append(resn)
            print(('Residue '+str(resn)+' is a PRO\n'))
            atomsasa[resn].append(sasa.get_atom_sasa()[
                                 resn][resobj.atom_index('O')])
            atomsasa[resn].append(0)
        else:
            for atomn in atoms:
                atomsasa[resn].append(sasa.get_atom_sasa()[
                                     resn][resobj.atom_index(atomn)])
    print('Atomic sasa:')
    print('betaresidue - NH - CO')
    pprint(atomsasa)
    # Put each stretch of at least 3 resi in dictionary
    beta_stretches = {}
    beta_stretches[pdbname.split('/')[-1]] = []
    i = 1
    for k, g in groupby(enumerate(betaresidues), lambda i_x: i_x[0]-i_x[1]):
        group = list(map(itemgetter(1), g))
        if not len(group) < 3:
            beta_stretches[pdbname.split('/')[-1]].append(group)
            i = i + 1

    # Calculate average sasa (NH and O) of each residue in each edge and put
stretch above threshold in new dict
    all_long_enough_exposed_edgestrands = {}
    all_long_enough_exposed_edgestrands[pdbname.split('/')[-1]] = []
    # Seperate proline stretches and non proline stretches. Doing this makes
it easier later
    proline_stretches_raw = {}
    remove_from_list = []
    for i in beta_stretches:
        for stretch in beta_stretches[i]:
            for pro in prolines:
                if pro in stretch:
                    proline_stretches_raw[pro] = []
                    proline_stretches_raw[pro].append(stretch)
                    remove_from_list.append(stretch)
    for i in beta_stretches:
```

```
        for j in remove_from_list:
            try:
                beta_stretches[i].remove(j)
            except:
                pass
    # Remove duplicates from proline stretches
    vals = []
    proline_stretches = {}
    for i in proline_stretches_raw:
        if not proline_stretches_raw[i][0] in vals:
            vals.append(proline_stretches_raw[i][0])
            proline_stretches[i] = []
            proline_stretches[i].append(proline_stretches_raw[i][0])
    print('\nBeta strands lacking prolines')
    pprint(beta_stretches)
    # Check average atomic sasa edgestrand to decide if it is exposed or not,
threshold is...
    for i in beta_stretches:
        for j in beta_stretches[i]:
            avg = []
            abego_stretch = get_abego(j)
            for res in j:
                avg.append(atomsasa[res])
            if np.mean(avg) >= exposed_threshold:
                # print(('Exposed edge avg',np.mean(avg),i,j))
                j.append('{0:.4g}'.format(np.mean(avg)))
                if 'A' in abego_stretch or 'E' in abego_stretch or 'G' in
abego_stretch:
                    if check_longest_beta(abego_stretch) == True:
                        all_long_enough_exposed_edgestrands[pdbname.split(
                            '/')[-1]].append(j)
                        # print(('Bulge present, long enough',i,j))
                    # else:
                    #     # print(('Bulge present, not long enough',i,j))
                else:
                    all_long_enough_exposed_edgestrands[pdbname.split(
                        '/')[-1]].append(j)
                    # print(('No bulge appending',i,j))
            elif np.mean(avg) <= exposed_threshold:
                j.append('{0:.4g}'.format(np.mean(avg)))
                # print(('This is a non exposed strand',np.mean(avg),i,j))

    print('\nBeta strands with prolines')
    pprint(proline_stretches)
    # Check average atomic sasa for edgestrands with PROLINE to decide if it
is exposed or not, threshold is...
    for i in proline_stretches:
        for j in proline_stretches[i]:
            avg = []
            abego_stretch = get_abego(j)
            for res in j:
```

```
                avg.append(atomsasa[res])
            if np.mean(avg) >= exposed_threshold:
                # print(('Exposed edge avg',np.mean(avg),i,j))
                j.append('{0:.4g}'.format(np.mean(avg)))
                if 'A' in abego_stretch or 'E' in abego_stretch or 'G' in
abego_stretch:
                    if check_longest_beta(abego_stretch) == True:
                        all_long_enough_exposed_edgestrands[pdbname.split(
                            '/')[-1]].append(j)
                        # print(('Pro and Bulge present,  long enough',i,j))
                    # else:
                    #     # print(('Pro and Bulge present,  not long
enough',i,j))
                else:
                    if check_longest_beta(abego_stretch) == True:
                        all_long_enough_exposed_edgestrands[pdbname.split(
                            '/')[-1]].append(j)
                        # print(('Pro but No bulge and EEE appending',i,j))
                    # else:
                    #     # print(('Pro and No bulge and no EEE
appending',i,j))
            elif np.mean(avg) <= exposed_threshold:
                j.append('{0:.4g}'.format(np.mean(avg)))
                # print(('This is a non exposed strand',np.mean(avg),i,j))

    global edges
    edges = all_long_enough_exposed_edgestrands
    print('\nExposed edgestrands:')
    pprint(edges)
    visualize_edgestrands_pymol()
    return all_long_enough_exposed_edgestrands


def visualize_edgestrands_pymol():
    '''Writes pymol script to visualize detected egdestrands'''
    colours = ['red', 'blue', 'yellow', 'violet', 'cyan',
               'salmon', 'lime', 'pink', 'slate', 'magenta', 'orange',
'marine',
               'olive', 'purple', 'teal', 'forest', 'firebrick', 'chocolate',
               'wheat', 'white', 'grey']
    count = 0
    for i in edges:
        with open(i+'.pml', 'w') as fout:
            fout.write('load '+name)
            fout.write('\nshow cartoon')
            fout.write('\nhide (h.)')
            fout.write('\ncolor gray90')
            for j, color in zip(edges[i], colours):
                avgatomsasa = edges[i][count][-1]
                fout.write('\nselect edge_'+str(count+1) +
                           '_AVGatomsasa'+avgatomsasa+'_'+i[:-4]+', i. ')
```

```
                for k in edges[i][count][:-1]:
                    fout.write(str(k)+'+')
                fout.write(' and '+i[:-4])
                count = count + 1
                fout.write('\ncolor '+color+', edge_'+str(count) +
                           '_AVGatomsasa'+avgatomsasa+'_'+i[:-4]+' and
'+i[:-4])
            fout.write(
                '\ncolor blue, elem N\ncolor red, elem O\ncolor yellow, elem
S')


def edgestrand_aligner(args, pose, name):
    '''Make edgestrand docks by aligning library of 2 stranded parallel and
anti
    parallel sheets'''
    def aligner(target, edge_dictionary, path):
        # Main alignement loop, worst code ever, need to clarify/rewrite
        for model in edge_dictionary:
            for i in range(len(edge_dictionary[model])):
                print('edge '+str(i+1), edge_dictionary[model][i][:-1])
                edge_dictionary[model][i] = edge_dictionary[model][i][:-1]
                window = 3
                step = 1
                num_frags_target = (
                    ((max(edge_dictionary[model][i]) -
min(edge_dictionary[model][i])) + 1) - window) / step + 1
                for lib in os.listdir(path):
                    strand_model = parser.get_structure(lib, path+'/'+lib)
                    print(path+'/'+lib)
                    for target_fragment in range(0,
int(num_frags_target*step), step):
                        resi_to_be_aligned_target = list(range(min(
                            edge_dictionary[model][i])+target_fragment,
min(edge_dictionary[model][i])+target_fragment+window))
                        # print resi_to_be_aligned_target
                        ca_T = []
                        for resi in resi_to_be_aligned_target:
                            ca_T.append(target[0]["A"][resi]["CA"])
                        # Start loop over strand fragments
                        # Make list with residues per chain
                        chs = ["A", "B"]
                        for ch in chs:
                            chain_atom_list = [int(str(res).split("
")[4].split("=")[
                                                                 1]) for model in
strand_model for chain in model if chain.get_id() == ch for res in chain]
                            num_frags_strand = (
                                chain_atom_list[-1] - chain_atom_list[0] + 1
- window) / step + 1
```

```
                            for strand_fragment in range(0,
int(num_frags_strand*step), step):
                                resi_to_be_aligned_strand = list(range(
                                    chain_atom_list[0]+strand_fragment,
chain_atom_list[0]+strand_fragment+window))
                                # print resi_to_be_aligned_strand
                                ca_S = []  # Make list in which CA atoms of
to be aligned residue are stored
                                for resi in resi_to_be_aligned_strand:

ca_S.append(strand_model[0][ch][resi]["CA"])

                                # Make superimposer object
                                super_imposer = Superimposer()
                                super_imposer.set_atoms(ca_T, ca_S)
                                super_imposer.apply(strand_model.get_atoms())
                                # save
                                io = PDBIO()
                                io.set_structure(strand_model)
                                outpdb =
'aln_'+name.split('/')[-1]+'-edge'+str(i+1)+'-'+str(resi_to_be_aligned_target
[0])+'-'+str(

resi_to_be_aligned_target[-1])+'_'+lib[:-8]+ch+'-'+str(resi_to_be_aligned_str
and[0])+'-'+str(resi_to_be_aligned_strand[-1])+'.pdb'
                                io.save(outpdb)
                                # Process outpdb so that only the non-aligned
chain is in the pdb, remove TER/END and rename new strand to chain A for
downstream processing
                                subprocess.check_output(
                                    "grep -v ' "+ch+" ' "+outpdb+" > temp.aln
&& mv temp.aln "+outpdb, shell=True)
                                subprocess.check_output(
                                    "sed '/TER/d' "+outpdb+" > temp && mv
temp "+outpdb, shell=True)
                                subprocess.check_output(
                                    "sed '/END/d' "+outpdb+" > temp && mv
temp "+outpdb, shell=True)
                                subprocess.check_output(
                                    "sed 's/ B / A /g' "+outpdb+" > temp &&
mv temp "+outpdb, shell=True)

    parser = PDBParser()
    if not args.database:
        print('\n location of database not specified, exiting\n')
        sys.exit(1)
    path = args.database
    if args.autodetect:
        edge_dictionary = new_get_edgestrands(pose, name)
        # get_beta_edgestrands() return edgedict and Pose in tuple, use only
index 0 --> this is the dictionary
```

```
        if args.bb_only:
            target_in = name
        else:
            target_in = args.s
        target = parser.get_structure(target_in, target_in)
        aligner(target, edge_dictionary, path)
    if args.edge:
        if args.bb_only:
            target_in = name
        else:
            target_in = args.s
        target = parser.get_structure(target_in, target_in)
        edge_dictionary = {}
        target_strand = args.edge.split('-')
        target_strand = [int(i) for i in
range(int(target_strand[0]),int(target_strand[1])+1)]
        assert int(target_strand[1]) > int(
            target_strand[0]), 'Start residue edge-strand must be smaller
than stop residue edge-strand'
        target_strand.append('dummy_value')
        edge_dictionary[target_in] = [target_strand]
        aligner(target, edge_dictionary, path)


def strip_side_chains(pose, name):
    '''Mutate all side chains to alanine'''
#    pose = pyrosetta.io.pose_from_pdb(args.s)
    for i in range(1, len(pose.sequence())+1):
        mut = pyrosetta.rosetta.protocols.simple_moves.MutateResidue(i,
'ALA')
        mut.apply(pose)
    pyrosetta.dump_pdb(pose, name)
    return pose


# main
pyrosetta.init('-ignore_unrecognized_res -mute all -beta_nov16
-renumber_pdb')
exposed_threshold = 2
pdb = args.s
line = '='
logname = len('Processing pdb: '+pdb)
print('\n')
print((logname*line))
print(('Processing pdb: '+pdb))
print((logname*line))
pose = pyrosetta.io.pose_from_file(pdb)

if args.autodetect and args.edge:
    print('\nUse either -autodetect or -edge flag but not both')
    sys.exit(1)
```

```
if args.autodetect:
    # if there are no strands at all in pose we can continue
    if len(check_for_strands(pose)) == 0:
        print(('no strands in pdb '+pdb))
    else:
        # If there are beta residues go through individual chains to find
edges
        print('\nFinding edgestrands in '+pdb)
        print('____________________________________')
        if len(check_for_strands(pose)) == 0:
            print(('no strands in pose '+pdb))
        else:
            if args.bb_only:
                print(
                    '\n-bb_only option passed: Converting pose to polyalanine
before finding edgestrands\n')
                name = 'polyala_'+pdb
                strip_side_chains(pose, name)
                switch_renumber(pose, name)
                edgestrand_aligner(args, pose, name)
            else:
                name = pdb
                switch_renumber(pose, name)
                edgestrand_aligner(args, pose, name)
if args.edge:
    if args.bb_only:
        print(
            '\n-bb_only option passed: Converting pose to polyalanine before
finding edgestrands\n')
        name = 'polyala_'+pdb
        strip_side_chains(pose, name)
        edgestrand_aligner(args, pose, name)
    else:
        name = pdb
        edgestrand_aligner(args, pose, name)

if args.fastrelax:
    if args.coordev:
        fastrelax(pose,args.coordev)
    else:
        fastrelax(pose,1.0)
```

minimize_strands.py

```
'''
Danny D. Sahtoe
dsahtoe@uw.edu, danny.sahtoe@gmail.com
University of Washington, Institute for Protein Design
Created Aug 2016
'''
import subprocess
```

```
import argparse
import sys
import pyrosetta

# Setup commandline options
parser = argparse.ArgumentParser()
parser.add_argument('-s',type=str, metavar='\b', default=None, help='Specify
target pdb (required)',required=True)
parser.add_argument('-strand',type=str, metavar='\b', default=None,
help='Strand pdb (required)',required=True)
parser.add_argument('-fix_target',action='store_true', help='Do not repack
target interface')
parser.add_argument('-repack_target_residues',type=str, metavar='\b',
default=None, help='comma separated list of residues to be repacked on target
interface')
parser.add_argument('-threshold',type=str, metavar='\b', default=None,
help='Edge-to-edge energies higher than this float will be filtered out
(required)',required=True)
args = parser.parse_args()


def fastrelax(target_pdb,pose,args,prefix):
    # somehow if not dumping pose but passing it, residueselectors remain
empty
    pdbinfo = pyrosetta.rosetta.core.pose.PDBInfo(pose)
    pose.pdb_info(pdbinfo)
    # dump_pdb(pose,args.strand+'_temp.pdb')
    # pose = pose_from_file(args.strand+'_temp.pdb')
    if len(pose.chain_sequence(2)) > 2:
        #Setup fast_relax
        scorefxn = pyrosetta.get_fa_scorefxn()
        scorefxn(pose)
        hbond_lr_bb_target = scorefxn.score_by_scoretype(target_pdb,
pyrosetta.rosetta.core.scoring.hbond_lr_bb)
        fast_relax = pyrosetta.rosetta.protocols.relax.FastRelax(2)
        fast_relax.set_scorefxn(scorefxn)
        #Setup residue selectors interface, Neighbors near chain_B, within 8
A, but not chain_B
        chain_B =
pyrosetta.rosetta.core.select.residue_selector.ChainSelector("B")
        chain_A =
pyrosetta.rosetta.core.select.residue_selector.ChainSelector("A")
        interfaceres_A =
pyrosetta.rosetta.core.select.residue_selector.NeighborhoodResidueSelector(ch
ain_B, 8, False )
        interfaceres_B =
pyrosetta.rosetta.core.select.residue_selector.NeighborhoodResidueSelector(ch
ain_A, 8, False )
        interface =
pyrosetta.rosetta.core.select.residue_selector.OrResidueSelector(interfaceres
_A,interfaceres_B)
```

```
        fixnotinterface =
pyrosetta.rosetta.core.select.residue_selector.NotResidueSelector(interface)
        scorefxn(pose)
        #Setup common min elements i.e. jump=True and always alllow bb and
sidechain min of strand.
        tf = pyrosetta.rosetta.core.pack.task.TaskFactory()

tf.push_back(pyrosetta.rosetta.core.pack.task.operation.IncludeCurrent())
        mm = pyrosetta.rosetta.core.kinematics.MoveMap()

mm.set_bb_true_range(pose.conformation().chain_begin(2),pose.conformation().c
hain_end(2))
        mm.set_jump(True)
        # make pack task according to flags
        try:
            if args.fix_target:

tf.push_back(pyrosetta.rosetta.core.pack.task.operation.OperateOnResidueSubse
t(pyrosetta.rosetta.core.pack.task.operation.PreventRepackingRLT(), chain_A,
False ))

tf.push_back(pyrosetta.rosetta.core.pack.task.operation.OperateOnResidueSubse
t(pyrosetta.rosetta.core.pack.task.operation.RestrictToRepackingRLT(),
chain_B, False ))
            elif args.repack_target_residues:
                repack_residues =
pyrosetta.rosetta.core.select.residue_selector.ResidueIndexSelector()
                for i in args.repack_target_residues.split(','):
                    repack_residues.append_index(int(i))
                chB_and_repackres =
pyrosetta.rosetta.core.select.residue_selector.OrResidueSelector(chain_B,repa
ck_residues)
                chA_fix =
pyrosetta.rosetta.core.select.residue_selector.NotResidueSelector(chB_and_rep
ackres)

tf.push_back(pyrosetta.rosetta.core.pack.task.operation.OperateOnResidueSubse
t(pyrosetta.rosetta.core.pack.task.operation.PreventRepackingRLT(), chA_fix,
False ))

tf.push_back(pyrosetta.rosetta.core.pack.task.operation.OperateOnResidueSubse
t(pyrosetta.rosetta.core.pack.task.operation.RestrictToRepackingRLT(),
chain_B, False ))

tf.push_back(pyrosetta.rosetta.core.pack.task.operation.OperateOnResidueSubse
t(pyrosetta.rosetta.core.pack.task.operation.RestrictToRepackingRLT(),
repack_residues, False ))
            else:

tf.push_back(pyrosetta.rosetta.core.pack.task.operation.OperateOnResidueSubse
```

```
t(pyrosetta.rosetta.core.pack.task.operation.PreventRepackingRLT(),
fixnotinterface, False ))

tf.push_back(pyrosetta.rosetta.core.pack.task.operation.OperateOnResidueSubse
t(pyrosetta.rosetta.core.pack.task.operation.RestrictToRepackingRLT(),
interface, False ))
        except:
            pass

        # apply settings and start min
        task = tf.create_task_and_apply_taskoperations( pose )
        fast_relax.set_movemap(mm)
        fast_relax.set_task_factory(tf)
        fast_relax.apply(pose)

        # Check whether hbond_lr_bb of complex is better than target alone by
certain amount (need to find objective number)
        # If it is dump the pdb after setting denovo strand to chain A and
target to chain B
        hbond_lr_bb_complex = scorefxn.score_by_scoretype(pose,
pyrosetta.rosetta.core.scoring.hbond_lr_bb)
        if hbond_lr_bb_complex-hbond_lr_bb_target <= float(args.threshold):
            #Switch chain order
            switch =
pyrosetta.rosetta.protocols.simple_moves.SwitchChainOrderMover()
            switch.chain_order("21")
            switch.apply(pose)
            pyrosetta.dump_pdb(pose, prefix+"_"+args.strand)
            with open(prefix+"_"+args.strand,'a') as f:
                f.write('delta_bb_hbond
'+str(hbond_lr_bb_complex-hbond_lr_bb_target))
            #setup directory and extract motif for grafting step
            subprocess.check_output('grep " A " '+prefix+"_"+args.strand+' >
motif_'+prefix+"_"+args.strand,shell=True)
            subprocess.check_output('mkdir '+prefix+'_'+args.strand[:-4]+';
mv '+prefix+'_'+args.strand+' '+prefix+'_'+args.strand[:-4]+'; mv
motif_'+prefix+'_'+args.strand+' '+prefix+'_'+args.strand[:-4],shell=True)
            subprocess.check_output('echo
'+str(hbond_lr_bb_complex-hbond_lr_bb_target)+'\t'+prefix+'_'+args.strand+'
>> hbond_score.list',shell=True)

def n1_cut():

pose.conformation().delete_residue_slow(pose.conformation().chain_begin(2))

def c1_cut():
    pose.conformation().delete_residue_slow(len(pose.sequence()))

def n2_cut():
```

```
pose.conformation().delete_residue_range_slow(pose.conformation().chain_begin
(2),pose.conformation().chain_begin(2)+1)

def n1c1_cut():

pose.conformation().delete_residue_slow(pose.conformation().chain_begin(2))
    pose.conformation().delete_residue_slow(len(pose.sequence()))

def c2_cut():

pose.conformation().delete_residue_range_slow(pose.conformation().chain_end(2
)-1,pose.conformation().chain_end(2))

pyrosetta.init()
target_pdb = pyrosetta.io.pose_from_file(args.s)
working_pose = pyrosetta.io.pose_from_file(args.s)
strand = pyrosetta.io.pose_from_file(args.strand)
pose = pyrosetta.Pose()
sfx_rep = pyrosetta.ScoreFunction()
sfx_rep.set_weight(pyrosetta.rosetta.core.scoring.ScoreType.fa_rep,0.55)

termini_cut = {'n1': [n1_cut],
        'c1': [c1_cut],
    'n2': [n2_cut],
    'n1c1': [n1c1_cut],
    'c2': [c2_cut]}

# For 3 residue strands, seperate dict, need to find better solution
termini_cut_mod = {'n1': [n1_cut],
        'c1': [c1_cut]}

subprocess.check_output('touch hbond_score.list',shell=True)

if args.strand.startswith('aln_'):
    endresi = subprocess.check_output('tail -1 '+args.strand,shell=True)
    endresi = int(endresi.split()[5])
    startresi = subprocess.check_output('head -1 '+args.strand,shell=True)
    startresi = int(startresi.split()[5])
    length = endresi - startresi + 1
    prefix='rlx_aln_untrimmed_'
    working_pose.append_pose_by_jump(strand,1)
    pose = working_pose.clone()
    # dont continue if the clash is severe
    if sfx_rep(pose) >= 5000:
        sys.exit(1)
    fastrelax(target_pdb,pose,args,prefix)
    if length > 3:
        for i in termini_cut:
            pose = working_pose.clone()
            prefix = 'rlx_aln_'+i
```

```
        for j in termini_cut[i]:
            j()
        fastrelax(target_pdb,pose,args,prefix)
else:
    for i in termini_cut_mod:
        pose = working_pose.clone()
        prefix = 'rlx_aln_'+i
        for j in termini_cut_mod[i]:
            j()
        fastrelax(target_pdb,pose,args,prefix)
```
